# Targeting autophagy reverses de novo resistance in homologous recombination repair proficient breast cancers to PARP inhibition

**DOI:** 10.1038/s41416-020-01238-0

**Published:** 2021-01-21

**Authors:** Ganesh Pai Bellare, Bhaskar Saha, Birija Sankar Patro

**Affiliations:** 1 Bio-Organic Division, Bhabha Atomic Research Centre, Trombay, Mumbai, Maharashtra 400085 India; 2 Homi Bhabha National Institute, Anushaktinagar, Mumbai, Maharashtra 400094 India

**Keywords:** Breast cancer, Cancer therapy

## Abstract

**Background:**

Poly(ADP-ribose) polymerase inhibitors (PARPi) target tumours defective in homologous recombination (HR). Most BRCA-wild-type (WT) HR-proficient breast cancers are intrinsically resistant to PARP inhibitors, e.g., talazoparib. We evaluated the role of autophagy in this de novo resistance and determined the underlying mechanism to overcome this.

**Methods:**

Autophagosome formation and autophagic flux were assessed by evaluating endogenous LC3-II levels and ectopic expression of EGFP-LC3 and mRFP-EGFP-LC3 in breast cancer cells. Autophagy-defective cells were generated by genetic depletion of BECN1, ATG5, p62/SQSTM1 and LAMP1 by using CRISPR-Cas9 double nickase system. The response of PARPi was evaluated in autophagy-proficient and -defective breast cancer cells and in xenograft SCID-mice model.

**Results:**

Pro-survival autophagy was significantly enhanced upon talazoparib treatment in BRCA-WT breast cancer cell lines. Autophagy-deficient cells were hypersensitive to talazoparib. Targeting autophagy synergistically enhanced the therapeutic efficacy of talazoparib in BRCA1-WT breast cancer cells in vitro and in vivo xenograft tumour mouse model. Mechanistically, autophagy inhibition by chloroquine promoted deleterious NHEJ mediated DSB-repair, leading to extensive genomic instability and mitotic catastrophe.

**Conclusions:**

Autophagy confers de novo resistance to PARP inhibitor, talazoparib. Autophagy inhibition improves the therapeutic outcome of PARPi treatment in preclinical mice model, bearing HR-proficient breast tumours, warranting its usage in the clinical settings.

## Background

Breast cancer is the most commonly diagnosed cancer and the leading cause of cancer-related mortality among women, worldwide.^1^ According to the International Agency for Research on Cancer, globally there were an estimated 2 million new cases and 0.6 million breast cancer-related deaths in 2018.^1^ Though the repertoire of detection techniques and therapeutic strategies have improved significantly over the past two decades, therapeutic resistance remains a major challenge in breast cancer management.

One of the relatively recent advancements in breast cancer therapeutics has been targeting poly (ADP-ribose) polymerases (PARPs). The involvement of PARPs in base excision repair (BER), homologous recombination (HR) repair and fork protection among other genome maintenance mechanisms emphasise the crucial role of PARPs in cells and presents itself as an attractive and viable druggable target.^2^ PARPs are frequently overexpressed in breast cancers, suggesting their importance in cancer cell survival.^3,4^ PARP inhibitors (PARPi) have shown promising single-agent anti-tumour efficacy in patients with BRCA-mutated breast cancer. Among the several clinically investigated PARP inhibitors (PARPi) e.g., olaparib, veliparib, rucaparib, niraparib, iniparib and talazoparib, FDA has approved talazoparib (Talzenna, Pfizer Inc.) and olaparib (Lynparza, AstraZeneca Inc.) for the treatment of BRCA-mutated breast and ovarian cancer patients with a deficient homologous recombination (HR) pathway through synthetic lethality.^5–9^ However, the success of PARPi therapy is severely limited to this small percentage of breast cancer patients with BRCA1/2 mutation (<10%).^10^ Moreover, PARPi therapy fails due to the development of acquired resistance in BRCA-mutant breast cancer patients. Of note, ~90% of breast cancers are BRCA-WT and homologous recombination proficient. Most of the BRCA-WT cancers are de novo resistant to PARPi treatment. This highlights a need for a therapeutic strategy that can be broadly applied across breast cancer patients and for tumours resistant to PARPi.

Several mechanisms of resistance to PARPi have been described. Reactivation of HR repair appears to be the underlying theme among all these mechanisms.^11^ It was shown that the intragenic deletion of BRCA2 and concomitant activation of HR was responsible for the limited sensitivity of cells to PARPi.^12^ The loss of poly(ADP-ribose) glycohydrolase (PARG) activity can restore the signalling functions of PARylation and restore HR in BRCA2-deficient cells.^13^ Post transcriptional regulation of PARG mRNA by Hu-antigen R (HuR) facilitates DNA repair and resistance to PARPi.^14^ Besides these mechanisms, the status of other DNA repair proteins also affects the response to PARPi. 53BP1 has been found to be one of the major regulators of PARPi response in patients, as it can regulate the choice of DNA repair pathways.^15,16^ Recently, it has been shown that targeting or downregulating HR is an efficient approach to sensitise BRCA-WT cancers (hepatocellular, ovarian, breast and laryngeal carcinoma) to PARP inhibition.^17–20^ Interestingly, a critical role for non-homologous end joining (NHEJ) was identified in inducing HR-deficient ovarian cancer cell death in response to PARPi treatment.^21^ It was also observed that the PARPi-mediated cell death was rescued by disabling NHEJ process.^21^ Since complex mechanisms of HR restoration confer resistance to PARP inhibitors, we hypothesised that simultaneous suppression of HR and elicitation of NHEJ may be a preferred strategy for sensitising BRCA-WT breast cancers.

Autophagy is one of the homoeostatic mechanisms aiding in the removal of dysfunctional and superfluous biomolecules and organelles from the cells under endogenous or exogenous stresses. Inhibition of autophagy leads to suppression of HR, due to CHK1 degradation and suppression of RAD51 recruitment in response to ionising radiation and etoposide treatment.^22^ Moreover, due to impaired HR, autophagy-deficient cells are hyperdependent on NHEJ for the repair of DNA double-strand breaks.^22^ Accumulated literature suggests that suppression of autophagy would lead to decreased HR and increased NHEJ. However, it is not yet understood whether autophagy activation may be linked to the underlying de novo resistance in BRCA-WT breast cancers to PARPi and whether targeting autophagy may benefit the PARPi-treatment outcome.

In this study, we present results that suggest that autophagy confers de novo resistance to talazoparib in BRCA-WT breast cancer cells. We show that the FDA approved anti-malarial drug, chloroquine, also an autophagy inhibitor, sensitises the effects of talazoparib in vitro and in vivo xenograft breast tumour model. We thereby establish an effective combinatorial approach for the treatment of BRCA-WT homologous recombination proficient breast cancers with homologous recombination proficiency.

## Methods

### Reagents and plasmids

Talazoparib (BMN673) was procured from ApexBio Technology (Houston, TX, USA). Chloroquine (CQ), MTT reagent, phalloidin conjugated with Alexa Fluor 488, Hoechst 33258, β-oestradiol and antibodies for p62/SQSTM1 (#P0067), LAMP1 (#L1418), β-actin (#A5441), 53BP1 (#MAB3802) were purchased from Sigma-Aldrich (St. Louis, MO, USA). Antibodies for BECN1 (#3495P), LC3A/B I/II (#12741), LC3B (#3868P), ATG5 (# 12994P), ATG7 (#8558P), ATG3 (#3145P), ATG16 (#8089P) were procured from Cell Signaling Technology (Danvers, MA, USA) and BRCA1 (#SC6954) and GAPDH (#SC47724) from Santa Cruz Biotechnology (Dallas, TX, USA). Anti-rabbit IgG and anti-mouse IgG secondary antibodies conjugated with horse-radish peroxidase for immunoblotting were procured from Roche (Basel, Switzerland). A secondary antibody conjugated with Alexa Fluor 488/594 for microscopy were purchased from Jackson Immunoresearch Laboratories (West Grove, PA, USA). Plasmids EGFP-LC3 (#24920) and mRFP-EGFP-LC3 (tf-LC3) (#21074) were from Addgene. Other fine chemicals were procured from Sigma-Aldrich (St. Louis, MO, USA).

### Cell lines and cell culture

Breast cancer cell lines MCF-7, MDA-MB-231, MDA-MB-453, T47-D, SKBR-3 and MDA-MB-468 were procured from National Centre for Cell Sciences, India. MCF-10A cells (immortalised normal breast epithelial cell line) were obtained from American Type Culture Collection (ATCC) and maintained as per the instructions given in ATCC. Cancer cells were maintained routinely in Dulbecco’s Modified Eagle’s medium with 10% foetal bovine serum and 1% penicillin–streptomycin solution in an incubator (95% relative humidity; 5% CO_2_; 37 °C). Experiments were performed with cells of fewer than eight passages after thawing the frozen stock. Testing for mycoplasma contamination was carried out at regular intervals. Cell line authentication was performed for the cell lines used in the study by short tandem repeat profiling.

### Clonogenic assay

Cells (500–750 cells/well) were seeded in a 6- or 12-well plate and treated post overnight incubation. Colony formation was allowed for 6–8 days and assessed after staining with 0.5% crystal violet solution.

### Generation of knockout/knockdown cell lines

The CRISPR-Cas9 double nickase plasmids for BRCA1, ATG5, LAMP1, BECN1 were purchased from Santa Cruz Biotechnology (Dallas, TX, USA). Control double nickase plasmid was used for control transfections. Sub-confluent MCF-7 cells were transfected using lipofectamine 2000 as per the manufacturer’s protocol. After puromycin selection, the knockout or depletion efficiency of the respective gene products was assessed by western blotting.

### Generation of EGFP-LC3 and mRFP-EGFP-LC3 (tf-LC3) expressing MCF-7 cells

Sub-confluent MCF-7 cells were transfected using lipofectamine 2000 and the EGFP-LC3 and mRFP-EGFP-LC3 plasmids. Stable transfectants were selected using G418 sulfate and expression of EGFP-LC3 and mRFP-EGFP-LC3 was assessed by microscopy.

### Cell viability assessment

Cells were seeded (5 × 10^3^ cells per well) in 96-well plates overnight. Cells were treated with different concentrations of the drugs for 48 h or 72 h, as indicated. Viable and metabolically active cells were assessed by MTT assay. MTT solution (0.5 μg/ml) was added (2 h). DMSO was added to solubilise formazan crystals, and absorbance was measured at 570 nm in a spectrophotometric microplate reader (BioTek, Germany).

### Immunoblotting and densitometry analysis

Briefly, 1.5 × 10^6^ cells were seeded overnight, treated and harvested. Following lysis and protein estimation, immunoblotting was performed, as described previously.^23^ Densitometric evaluation of the blots was carried out using ImageJ (v1.51j8).

### Flow cytometric analyses

Sub-G1 assay for apoptosis detection and cell cycle analyses were carried out, as described previously.^24^ The analysis of results was carried out by using FlowJo software.

### Drug interaction study

The interaction between the effects of talazoparib and chloroquine, in clonogenic and sub-G1 assays, was assessed by CompuSyn software based on Chou–Talalay equation.^24^ Combination index (CI) is a parameter giving a quantitative result to analyse the interaction between drugs to produce an effect in the system being studied. The CI equation offers a quantitative definition for additive effect (CI = 1), synergism (CI < 1), and antagonism (CI > 1) in drug combinations.

### Immunofluorescence microscopy

Cells (7.5 × 10^4^ cells per well) were seeded in a six-well plate containing glass coverslips. Cells were treated and for the indicated time and fixed with 2.5% paraformaldehyde for 20 min. Cells were permeabilised using phosphate-buffered saline (PBS) containing 0.1% Triton X-100 (PBST) for 10 min and blocked with bovine serum albumin (BSA; 5% in PBST). Antibodies specific for α-tubulin, 53BP1, LC3-II, p62/SQSTM1 or phalloidin conjugated with Alexa Fluor 488 were used. This was followed by incubation with secondary antibody conjugated with Alexa Fluor 488/594 in 2.5% BSA in PBST (2 h). Samples were washed, dried and mounted onto glass slides using 80% glycerol with Hoechst 33258. Slides were observed under a confocal laser scanning microscope (LSM 780, Carl Zeiss, Germany). Image analysis was performed using Zeiss Zen software.

### Mice and tumour injection

Healthy female SCID mice (6–9-weeks old, ~26–32 g average body weight) were purchased from Advanced Centre for Treatment, Research and Education in Cancer (ACTREC, Navi Mumbai, India). Approval for followed experimentation and the protocols in the current study was obtained from the Institutional Animal Ethics Committee (IAEC) of Bhabha Atomic Research Centre (Approval number- BAEC/10/16). Mice were housed in standard individually ventilated polycarbonate shoebox cages (3–4 mice per cage), received standard diet and care in accordance with the guidelines of the IAEC. SCID mice were kept in a germ-free aseptic room, and all the treatments were given in a laminar hood. Exponentially growing MCF-7 cells (5 × 10^6^ cells per 100 µl diluted DMEM (1:1 dilution in PBS) was injected into the right flank of the mice. β-oestradiol (200 ng/0.1 ml PBS) supplementation was given every alternate day by subcutaneous injection.

### Mice treatment with talazoparib and CQ

Treatment began 15 days post tumour implantation consequent to palpable tumour formation. Mice were randomised into different groups prior to treatment (*n* = 6 for each group). According to the treatments, mice groups were named as: (1) vehicle alone (100 μl, 0.5% DMSO in PBS), (2) talazoparib (2 mg/kg/day; 100 μl; 0.5% DMSO in PBS), (3) CQ (50 mg/kg/day; 100 μl PBS) and (4) talazoparib plus CQ. Vehicle and both the drugs (talazoparib and chloroquine) were administered to the respective groups of mice through oral gavage (*per os*) on alternate days for 30 days.

### Tumour volume measurement

Tumour volume (TV) was evaluated by measuring the perpendicular diameter axes of the tumour with Vernier callipers (Axes labels- ‘a’, long axis, and ‘b’, short axis). TV was calculated using the formula (V) = (a × b^2^)/2. Relative TV with respect to initial TV was calculated and plotted graphically. Growth curves were plotted as a function of time.^17,25^ Two independent persons, who were unaware of the treatment protocols, measured the tumour volume in the mice. The macroscopic examination was carried out at the end of the experiments to evaluate the treatment-related organ toxicity, after euthanising the animals by CO_2_ asphyxiation in compliance with the standard procedure approved by the IAEC. Briefly, CO_2_ cages were used to steadily replace the air with CO_2_ (5–20% cage air with CO_2_ per minute) for 8 minutes. After CO_2_ treatment, mice were returned to ambient air for 15 min to ensure no recovery.

### Toxicity studies in mice

Female non-tumour-bearing SCID mice (8–10-weeks old, 23–28 g average body weight) were randomly segregated into four groups before the start of the experiment (*n* = 3 in each group). The mice were treated as per the protocol established for tumour-bearing mice described earlier. Body weights of individual mice were recorded using an electronic balance every alternate day. After 15 doses of the drug/s, the mice were sacrificed following euthanasia, as previously mentioned. Blood was collected from the animal by cardiac puncture using a syringe needle, coagulated and the serum isolated was analysed for biochemical parameters e.g., serum glutamic oxaloacetic transaminase (SGOT, AST—Aspartate transaminase), serum glutamic pyruvic transaminase (SGPT, ALT—alanine transaminase), alkaline phosphatase (ALP) and creatinine levels. All the biochemical measurements were carried out using an autoanalyser (Rx Daytona, Randox, Crumlin county, Antrim, UK). Liver, spleen and kidneys were harvested and processed for histopathology studies. Images were analysed for changes in the tissue architecture with treatments. For histopathological studies, organ tissue samples were fixed in Bouin’s fixative, and stained with haematoxylin–eosin (H–E) after preparation of 5-μm-thick paraffin sections. The stained slides were observed under a bright-field light microscope (Axioskop II Mot plus, Carl Zeiss, Germany), and images were captured using a camera. All the biochemical and histopathological analyses were blinded and carried out independently by the biochemist and pathologist.

### HPLC analysis of talazoparib concentrations in serum

Mice were given vehicle or talazoparib (2 mg/kg body weight) *per os* and serum was collected after 2, 6 and 24 h time points. Talazoparib concentrations in serum were assessed by HPLC analysis as per the reported protocol.^26^

### Statistical analyses

At least three independent experiments were carried out and values presented as mean ± SEM. Statistical analysis was performed using GraphPad Prism 5.0. Two-tailed, unpaired, Student’s *t* test or ANOVA with Tukey post-hoc analysis, was carried out, wherever necessary, to test the statistical significance of the data presented. A value of *P* < 0.05 was considered significant. For animal experiments, all animal studies were conducted using three to six animals per group for each experiment. The statistical significance was determined by using Student’s *t* test. Data from one independent experiment are shown.

## Results

### BRCA-WT homologous proficient breast cancer cells and tumour xenograft are resistant to talazoparib treatment

**Fig. 1:**
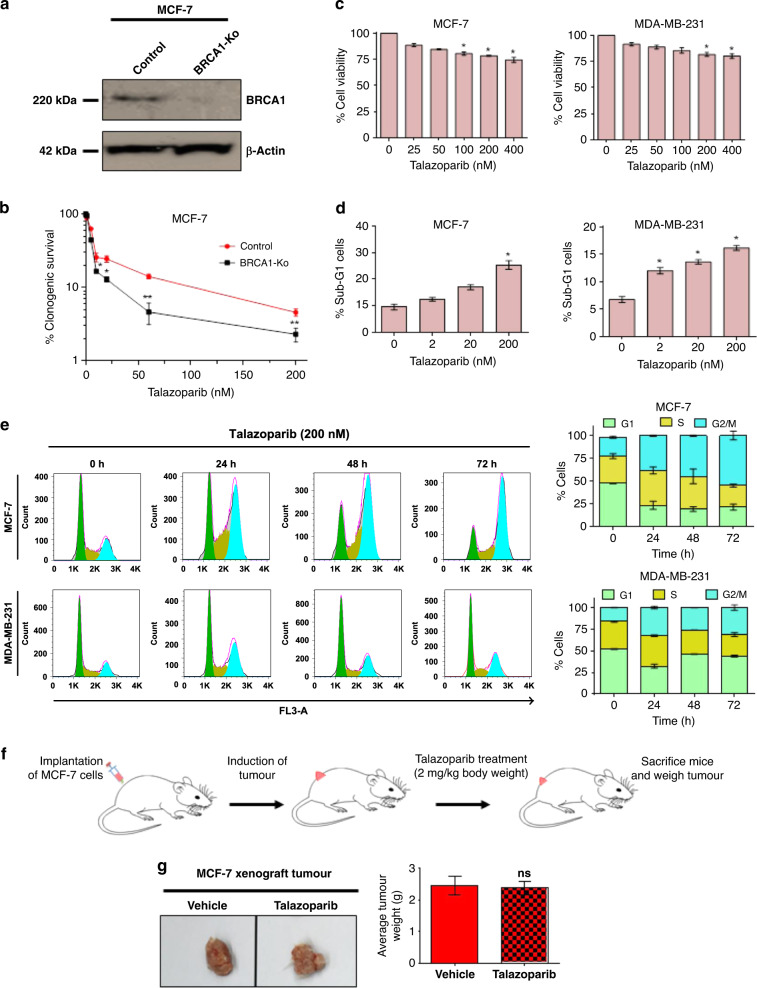
BRCA-WT HR-proficient breast cancer cells are de novo resistant to talazoparib monotherapy. **a** Protein level of BRCA1 protein in control (BRCA1-WT) and BRCA1-KO MCF-7 cells was assessed by western blotting. **b** Control (BRCA1-WT) and BRCA1-KO MCF-7 cells were treated with talazoparib (0–200 nM) for 6–8 days, and colony-forming ability was assessed by clonogenic assay. **c** MCF-7 and MDA-MB-231 cells were treated with talazoparib for 48 h with the indicated concentrations, and cell viability was assessed by MTT assay. **d** MCF-7 and MDA-MB-231 cells were treated with talazoparib for 72 h with the indicated concentrations, and the apoptotic sub-G1 population was assessed by flow cytometry. **e** MCF-7 and MDA-MB-231 cells were incubated with 200 nM talazoparib for 24, 48 and 72 h. Cell cycle analysis was performed by flow cytometry, and the cell cycle distribution was analysed by FlowJo software. **f** Schema for implantation of MCF-7 tumour xenograft, treatment regimen and the determination of tumour burden in SCID mice. **g** SCID mice were treated as per the schema, and tumour burden was measured after 15 doses of talazoparib (*per os*, 2 mg/kg body weight, *n* = 6 per group). All the determinations except the animal experiment were made from three to five experiments, and the values indicated are mean ± S.E.M. *P < 0.05, **P < 0.01. ns not significant compared to respective vehicle control.

Initially, BRCA1-KO MCF-7 cells were generated using CRISPR-Cas9 approach (Fig. 1a), and synthetic lethal interaction of these cells was validated in response to PARPi treatment. Our clonogenic assay results showed that BRCA1-KO cells vis-à-vis BRCA-WT cells, were highly sensitive to talazoparib (0–200 nM, Fig. 1b), suggesting HR-mediated de novo resistance in BRCA-WT cells. MTT assay results also showed that the cell viability of BRCA-WT breast cancer cells (MCF-7 and MDA-MB-231) was minimally affected in response to short-term treatment of talazoparib (0–400 nM; 48 h) (Fig. 1c). Considering a recent report that short-term MTT assay may not induce viability loss,^27^ we performed sub-G1 analysis to evaluate apoptosis induction upon talazoparib treatment. We observed that although talazoparib treatment induced apoptosis in a dose-dependent manner in both the cell lines tested, only ~25% and 16% of sub-G1 population were induced in MCF-7 and MDA-MB-231 cells, respectively, in response to the highest tested concentration of talazoparib (200 nM; 72 h) (Fig. 1d). We next investigated the effects of talazoparib on the cell cycle distribution. Cell cycle analysis by flow cytometry revealed that both the cell lines were arrested at the G2/M phase upon talazoparib treatment (Fig. 1e). G2/M phase arrest was prominent in p53-WT MCF-7 cells compared to the p53-mutant MDA-MB-231 cell line. Sub-G1 data corresponding to the cell cycle analysis data is also shown in Supplementary Fig. S1A, B. We then sought to evaluate the efficacy of talazoparib on BRCA-WT breast tumour xenograft in the preclinical SCID-mice model. We observed that after talazoparib treatment (2 mg/kg body weight; 15 doses; each dose on every alternate day) (Fig. 1f), the tumour burden in mice was comparable with that of the vehicle-treated group with no significant change in the tumour weight (Fig. 1g). Collectively, talazoparib treatment alone was found to be ineffective in BRCA-WT homologous recombination proficient breast cancer cell lines and xenograft breast tumour due to de novo or intrinsic resistance. Additionally, the percent viability of non-malignant MCF-10A cell line treated with different concentrations of talazoparib was tested and found to be greater than that of MCF-7 cells at the same concentrations, indicating the greater tolerance of talazoparib doses by normal cells (Supplementary Fig. S2A). Sub-G1 analysis for apoptosis induction also indicated that compared to MCF-7 cells, MCF-10A cells had significantly lower apoptosis induction at 48 h and 72 h at the tested concentrations of talazoparib (Supplementary Fig. S2B, C).

Of note, we carried out all the short-term biological assays (0–72 h) at a higher concentration of talazoparib (200 nM), where all the cellular and molecular events are expected to occur at higher levels. Since, the clinically relevant concentration of talazoparib is 25–50 nM,^28^ most of the key experiments were also carried out at 50 nM talazoparib.

### Talazoparib elicits stabilisation of autophagy initiation factors and induces autophagosome formation in BRCA-WT breast cancer cells

**Fig. 2:**
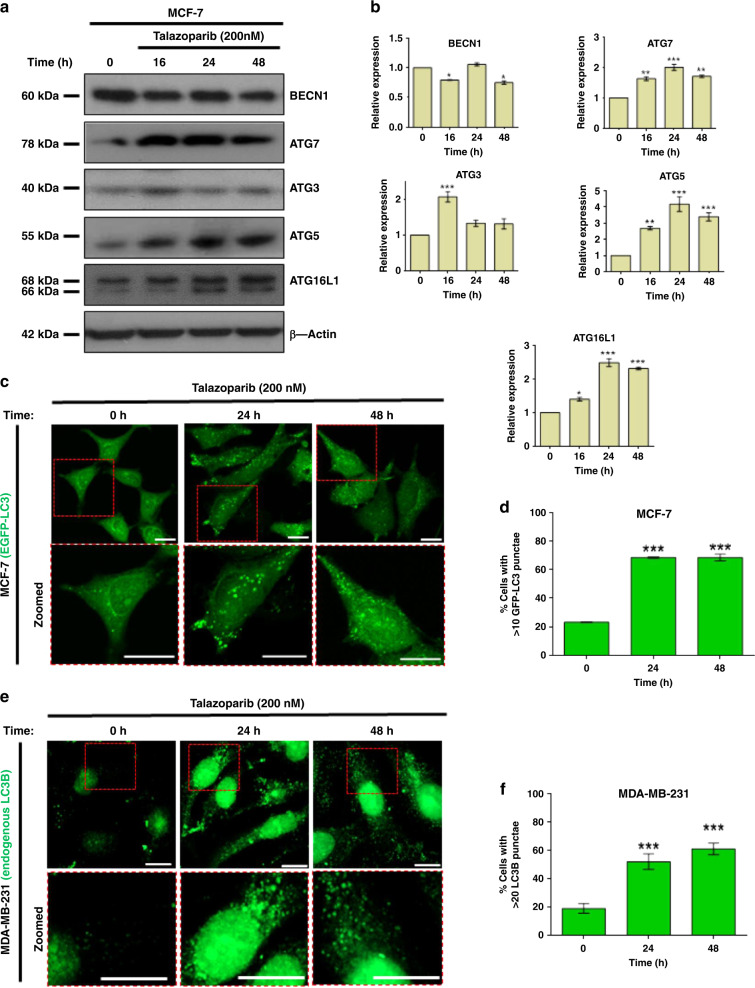

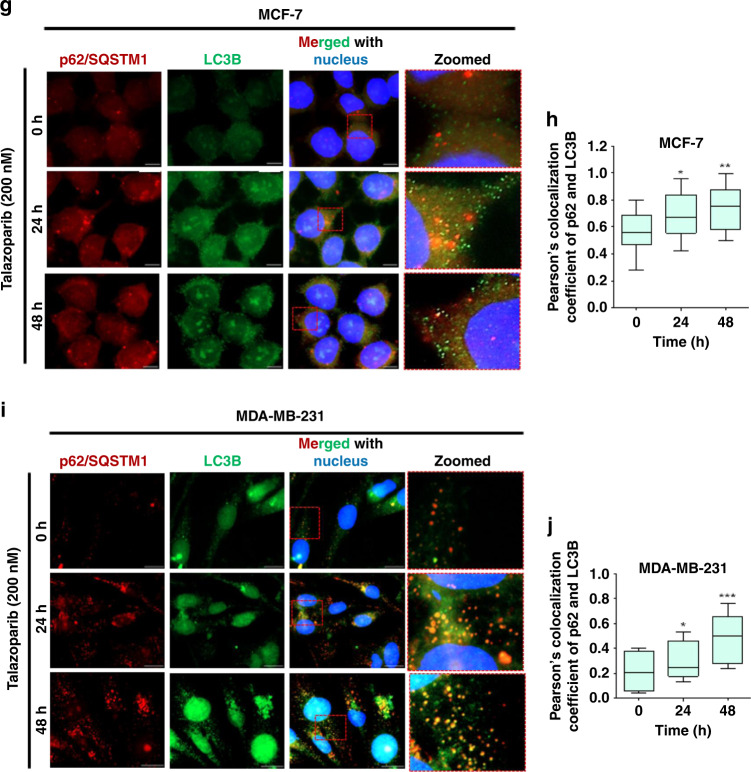
(continued)

Autophagy has been reported to confer intrinsic resistance to several chemotherapeutics and radiation therapy.^29^ However, it is not yet known whether autophagy is linked to de novo resistance in BRCA-WT breast cancer to PARPi treatment. To understand this, we sought to know whether autophagy was induced in response to PARPi treatment. In this regard, we observed that the levels of proteins essential for autophagy initiation i.e., ATG3, ATG5, ATG7 and ATG16L1 were significantly enhanced in a time-dependent manner upon talazoparib treatment (200 nM, 0–48 h) in MCF-7 cell line (Fig. 2a, b). LC3-II protein is a part of the autophagosomal membrane and forms distinct punctae upon autophagy induction. To this end, we generated MCF-7 cells stably expressing EGFP-LC3 and observed a significant increase in the level of EGFP-LC3 punctae in a time-dependent manner upon talazoparib treatment (Fig. 2c, d). In addition, by immunofluorescence microscopy, we also observed an increase in the endogenous LC3B punctae in MDA-MB-231 cells (Fig. 2e, f) in a time-dependent manner. Poly-ubiquitinated cargo for autophagic degradation is recognised by p62/SQSTM1 and loaded into LC3-II decorated autophagosomes.^30^ In this regard, we observed a time-dependent increase in co-localisation of p62/SQSTM1 with LC3 in both MCF-7 (Fig. 2g, h) and MDA-MB-231 cells (Fig. 2i, j), upon talazoparib treatment (200 nM). These results suggested that talazoparib treatment induces stabilisation of autophagy initiation factors and autophagosome formation in breast cancer cells.

### Talazoparib enhances autophagic flux in breast cancer cells

**Fig. 3:**
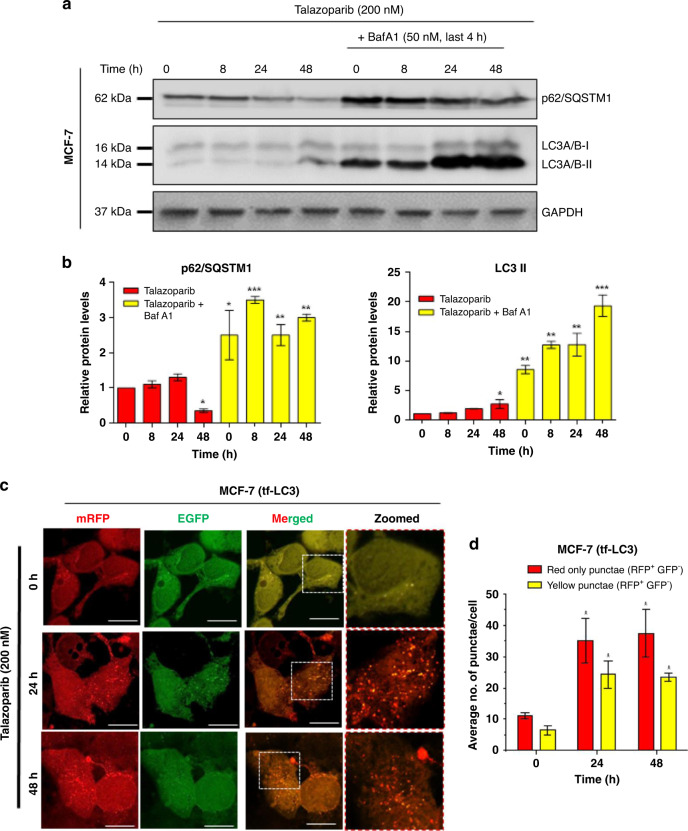
Autophagic flux was enhanced in breast cancer cell lines in response to talazoparib. **a**, **b** MCF-7 cells were treated with talazoparib (200 nM) for the indicated time, and immunoblot analysis was performed to assess autophagic flux as measured by LC3-II and p62 accumulation in the presence of BafA1 (50 nM for the last 4 h of incubation). GAPDH was used as the loading control. **c**, **d** MCF-7 cells stably expressing mRFP-EGFP-LC3, were treated with talazoparib (200 nM) for 24 h and 48 h, and autophagic flux was assessed by confocal microscopy. Red only (RFP^+^ GFP^−^) and yellow punctae (RFP^+^ GFP^+^) in cells were manually quantified from the merged image, and the average number of red only punctae (RFP^+^ GFP^−^) and yellow punctae (RFP^+^ GFP^+^) per cell are plotted. Scale bar: 20 μm. In western blots, quantification of the band intensity was done by using ImageJ (v1.51j8). Band intensities of the treatment groups were normalised by considering the value of vehicle control as 1. All the determinations were made from three to five independent experiments, and the values indicated are mean ± S.E.M. For **b** *P < 0.05, **P < 0.01 and ***P < 0.001 compared to respective vehicle control. For **d** *P < 0.05 and ***P < 0.001 compared to the respective parameters in 0 h.

Autophagic flux is a measure of completion of autophagy by accomplishing fusion of autophagosomes with lysosomes. The autophagic flux is essential for the effective outcome leading to cell survival. Certain drugs induce autophagy initiation alone, but the autophagic flux and completion of the process are not achieved.^31^ In such a scenario, cells may not derive survival advantages. We, therefore, sought to understand whether talazoparib induced autophagosomes (Fig. 2) may be subsequently cleared through the autophagic flux in BRCA-WT cells. We performed the standard assay for understanding autophagic flux, involving bafilomycin A1 (BafA1)-mediated inhibition of lysosomal acidification and late-stage inhibition of autophagy. We observed a time-dependent enhanced accumulation of the LC3-II in response to talazoparib in the presence of BafA1, in comparison to talazoparib treatment in the absence of BafA1 (Fig. 3a, b). We also observed the degradation of p62 in response to talazoparib, which was blocked in the presence of BafA1, due to blockade of autophagic degradation by lysosomes (Fig. 3a, b). Further, talazoparib-induced clearance of p62/SQSTM1 in MCF-7 cells was also blocked by chloroquine (CQ) (Supplementary Fig. S3A, B). CQ is an inhibitor of the autophagosome and lysosome fusion process.^32,33^ Together, our results showed an increase in autophagic flux, in a time-dependent manner, in response to talazoparib. A comparable autophagic flux was also induced in non-malignant MCF-10A cells upon talazoparib treatment (200 nM) (Supplementary Fig. S3C, D).

In order to further validate autophagic flux in PARPi treated BRCA-WT breast cancer cells, a pH-sensitive mRFP-EGFP-LC3 tandem fluorescence probe was employed in our study.^34^ Mature autophagosomes with mRFP-EGFP-LC3 forms yellow puncta, due to red and green fluorescence, while autophagolysosomes show only red puncta, as fluorescence of EGFP in mRFP-EGFP-LC3 is quenched in the acidic environment of autophagolysosomes.^34^ The microscopy results with MCF-7 cells, stably expressing exogenous mRFP-EGFP-LC3, showed that red only (RFP^+^ GFP^−^) and yellow (RFP^+^ GFP^+^) punctae were profusely enhanced (24 and 48 h) in response to talazoparib treatment (200 nM; Fig. 3c, d). Quantification of tf-LC3 punctae was carried out by following the previous report.^35^ Presence of red only (RFP^+^ GFP^−^) punctae suggested acidification of autophagolysosomes and hence active autophagic flux in PARPi treated BRCA-WT MCF-7 cells. Similar results were also obtained in BRCA1-WT MCF-7 cells in response to the lower and clinically relevant concentrations of talazoparib (50 nM; Supplementary Fig. S4A, B). In order to evaluate whether BRCA1 may regulate autophagy induction in breast cancer cells, BRCA1-KO MCF-7 cells were also used. In comparison to BRCA1-WT cells, a similar level of red only (RFP^+^ GFP^−^) and yellow (RFP^+^ GFP^+^) punctae in mRFP-EGFP-LC3 was observed in BRCA1-KO MCF-7 cells in response to talazoparib (50 nM; Supplementary Fig. S4A, B). In the presence of CQ, quenching of green fluorescence of mRFP-EGFP-LC3 was suppressed, indicating a robust autophagosome formation and autophagic flux in both control and BRCA1-KO cells. Together, our results suggested that talazoparib treatment at tested concentrations led to significant enhancement of autophagic flux process in both control (BRCA1-WT) and BRCA1-KO MCF-7 cells.

### Genetic depletion of autophagic components leads to sensitisation of BRCA-WT cells to talazoparib

**Fig. 4:**
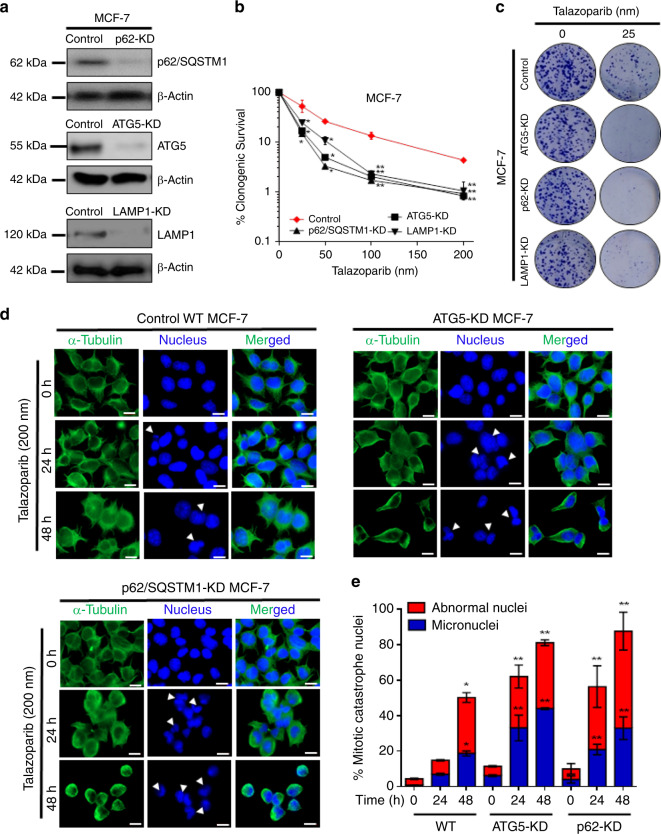
Autophagy confers intrinsic resistance to PARP inhibitor in BRCA1-WT HR-proficient breast cancer cells. **a** p62/SQSTM1, ATG5 and LAMP1 was depleted by using CRISPR-Cas9 double nickase system in BRCA1-WT control MCF-7 cells. The levels of these proteins were assessed by western blot analysis. **b**, **c** Control MCF-7 and p62/SQSTM1, ATG5 and LAMP1-depleted MCF-7 cells were treated with indicated concentrations of talazoparib for 6–8 days, and their clonogenic potential was assessed. Representative images of clonogenic assay results are shown. **d** Control MCF-7 and p62/SQSTM1, ATG5 and LAMP1-depleted MCF-7 cells were treated with talazoparib for 24 h and 48 h, and mitotic catastrophe events were assessed by confocal microscopy. Scoring of events was done for at least 100 cells in each condition. Experiments were repeated at least thrice. Scale bar: 20 μm. Arrowheads in the microscopic images indicate the nuclei with mitotic catastrophe. All the determinations were made from three to five experiments, and the values indicated are mean ± S.E.M. For **b** *P < 0.05 and **P < 0.01 compared to respective vehicle control. For **e** *P < 0.05 compared to untreated control in WT cells, **P < 0.01 compared to respective treatment and time point in WT cells.

We generated BECN1 knockout in MCF-7 cells by using CRISPR-Cas9 double nickase system (Supplementary Fig. S5A). BECN1 is an important protein known to regulate autophagy.^36^ Intriguingly, our results showed that sensitivity of BECN1-KO cells to talazoparib was similar to that of control cells (Supplementary Fig. S5B). In addition, we observed a robust induction of EGFP-LC3 punctae in BECN1-KO MCF-7 cells stably expressing EGFP-LC3, in response to talazoparib treatment similar to that in control MCF-7 cells (Supplementary Fig. S5C, D). Moreover, talazoparib treatment also enhanced red only (RFP^+^ GFP^−^) punctae (i.e., autophagolysosomes) and yellow (RFP^+^ GFP^+^) punctae (i.e., autophagosomes) formation in mRFP-EGFP-LC3 expressing BECN1-KO breast cancer cells similar to that seen in control MCF-7 cells (Supplementary Fig. S5E, F). This suggests that BECN1 is dispensable for PARPi-mediated autophagy activation in MCF-7 breast cancer cells. The dispensable role of BECN1 in non-canonical autophagy induction in MCF-7 cells has been reported earlier.^37^ Since ATG5, p62/SQSTM1 and LAMP1 play a pivotal role during initiation, maturation and completion process of autophagy, respectively,^38^ we knocked-down these genes individually in MCF-7 cells by CRISPR-Cas9 double nickase system (Fig. 4a). Further, p62/SQSTM1-KD cells were unable to form mature mRFP-EGFP-LC3 puncta in response to talazoparib, confirming autophagy deficiency in these cells (Supplementary Fig. S6). Our clonogenic assay results revealed that these autophagy-deficient BRCA-WT cells, ATG5-KD, p62/SQSTM1-KD and LAMP1-KD, were highly sensitive to talazoparib treatment (Fig. 4b, c). The sensitivity of these autophagy-deficient cells was even better than BRCA1-KO cells (Fig. 1b).

Reports suggest that PARP inhibitors induce replication stress and enhance genomic instability.^39,40^ It has also been established that PARP inhibitor toxicity in HR-deficient cancer cells is in part due to the progression through mitosis with accumulated genomic aberrations.^41^ Since autophagy is also known to play a vital role in genome maintenance,^42,43^ we sought to know whether de novo resistance to PARPi could be linked to autophagy-mediated genome maintenance in BRCA1-WT breast cancer cells. In this regard, our results indicated that mitotic catastrophe (multi-lobular, multinucleated and chromatin bridges; Supplementary Fig. S7), consequent to genomic instability, was significantly higher in autophagy-deficient MCF-7 cells (ATG5-KD and p62/SQSTM1-KD) *vis-à-vis* autophagy-proficient (control MCF-7) cells in response to talazoparib (200 nM; 24 h and 48 h) (Fig. 4d, e). Genomic instability and the consequent mitotic catastrophe were significantly higher in BRCA1-KO and ATG5-KD MCF-7 cells than control MCF-7 cells, even upon low concentrations of 50 nM talazoparib alone treatment, indicating genomic instability as an important cell death mechanism and mitotic catastrophe as a major mode of cell death in BRCA1 and ATG5-deficient cells (Supplementary Fig. S8A, B). Collectively, the results suggested a pivotal role for autophagy in de novo resistance and genome maintenance in BRCA-WT breast cancer cells in response to PARPi treatment.

### Pharmacological inhibition of autophagy by chloroquine sensitises BRCA-WT breast cancer cells to PARPi by promoting NHEJ

**Fig. 5:**
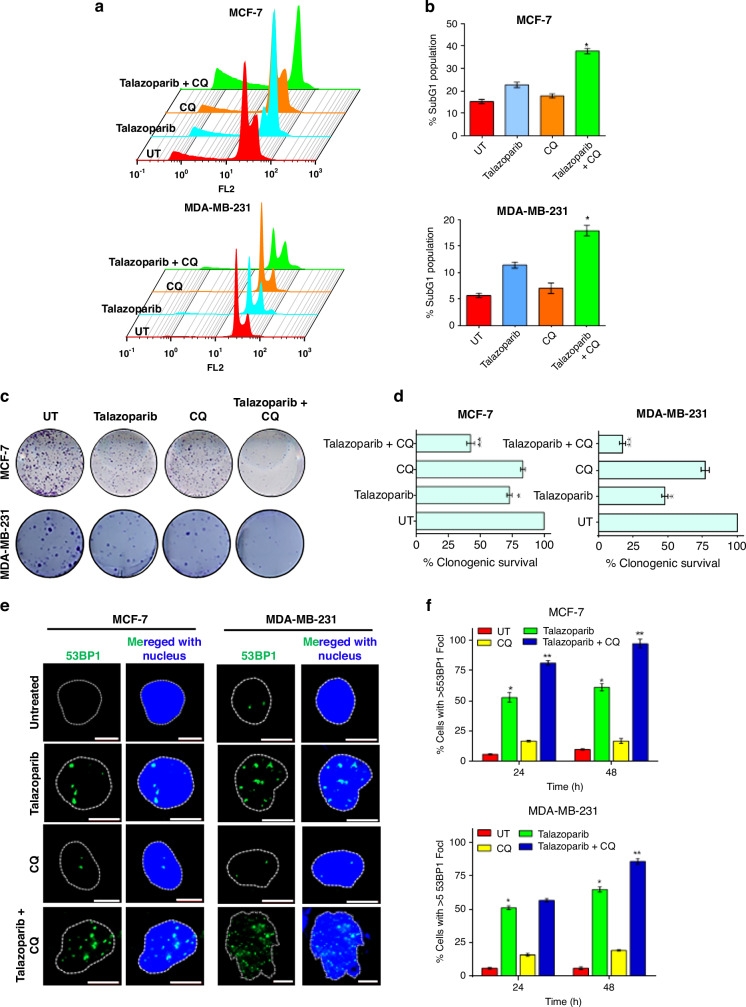

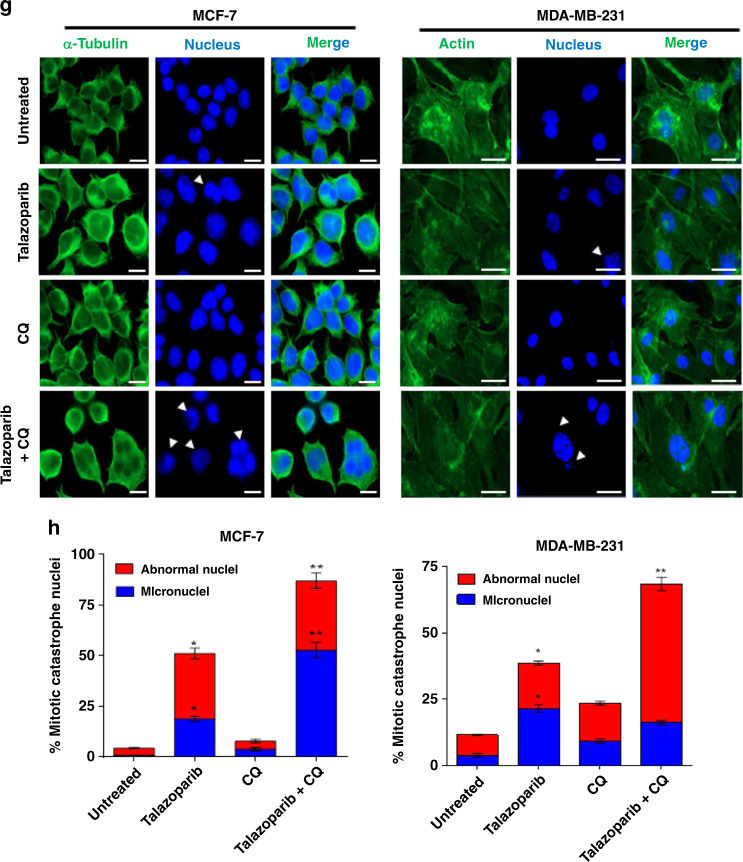
Pharmacological inhibition of autophagy sensitises BRCA-WT breast cancer cells by promoting NHEJ. **a**, **b** MCF-7 and MDA-MB-231 cells were treated with talazoparib (200 nM), CQ (10 μM) or combination of talazoparib and CQ for 72 h, and cell death was assessed by sub-G1 analysis using flow cytometry. Quantification was done using FlowJo software. **c**, **d** MCF-7 and MDA-MB-231 cells were treated with talazoparib (25 nM), CQ (2.5 μM) or a combination of talazoparib and CQ and their clonogenic potential was assessed. **e**, **f** MCF-7 and MDA-MB-231 cells were treated with talazoparib (200 nM), CQ (10 μM) or combination of talazoparib and CQ for 48 h, and the 53BP1 foci formation was assessed by immunofluorescence assay. The samples were analysed by confocal microscopy, and quantification was done by manually counting cells with >5 foci. **g**, **h** MCF-7 and MDA-MB-231 cells were treated with talazoparib (200 nM), CQ (10 μM) or a combination of talazoparib and CQ for 48 h, and mitotic catastrophe events were assessed by confocal microscopy by staining the nucleus and alpha-tubulin or actin in MCF-7 and MDA-MB-231 cells, respectively. Quantification was done by manual counting of mitotic catastrophe nuclei. Scale bar: 20 μm. All the determinations were made from three to five experiments, and the values indicated are mean ± S.E.M. *P < 0.05 com*p*ared to respective vehicle control, **P < 0.05 compared to respective talazoparib alone treatment.

Many of the macroautophagy genes are essential in early development, and defect in autophagy is not tolerated.^44^ Genetic defects in autophagy proteins are a rare occurrence in breast cancer patients.^45^ We, therefore, sought to use a pharmacological inhibitor to target autophagy-mediated de novo resistance in BRCA-WT breast cancer to PARPi. Chloroquine is an anti-malarial drug, repurposed as an autophagy inhibitor for cancer therapy. It is currently going through multiple clinical trials. It has been shown to inhibit autophagic flux by decreasing autophagosome–lysosome fusion in addition to being a lysosomotropic agent.^32,33^ As shown in Fig. 5a–d, cotreatment of CQ significantly sensitised the induction of apoptosis and also reduced the colony formation in BRCA-WT (MCF-7 and MDA-MB-231) cells treated with talazoparib. In corroboration with earlier reports, MDA-MB-231 cells, being triple-negative breast cancer cell type and having characteristics similar to BRCA-mutant cancers,^46^ its sensitivity to talazoparib may be comparatively higher than MCF-7 cells. The combinatorial effects of talazoparib and CQ were found to be synergistic in sub-G1 and clonogenic assay, as per the combination index assessed by using CompuSyn Software based on Chou–Talalay equation (Supplementary Fig. S9A–D; data not shown for clonogenic assay). Other autophagy inhibitors like 3-methyladenine also yielded similar results in sub-G1 assay (Supplementary Fig. S10). In ovarian cancer cells, it has been shown that NHEJ repair process plays a critical role in PARPi-mediated synthetic lethality in HR-deficient cells. Further, we wanted to know whether autophagy inhibition, already known to reduce HR,^21^ can enhance NHEJ process to sensitise BRCA-WT cells to PARPi. Intriguingly, we observed that talazoparib induced extensive 53BP1 foci formation, known to promote NHEJ,^47^ and this was enhanced by cotreatment with CQ in two different BRCA-WT cells tested (Fig. 5e, f). In corroboration with this and earlier results (Fig. 4), CQ also significantly enhanced mitotic catastrophe consequent to genomic instability in BRCA-WT cell lines in response to talazoparib treatment (Fig. 5g, h). We also observed similar enhancement in 53BP1 foci formation and mitotic catastrophe at lower talazoparib concentration in MCF-7 and MDA-MB-231 cells (50 nM; Supplementary Fig. S11A, D and S12A–D). Interestingly, talazoparib-induced 53BP1 foci formation was significantly enhanced in HR-deficient BRCA1-KO and ATG5-KD cells vis-à-vis control MCF-7 cells (50 nM; Supplementary Fig. S11A–D). Collectively, our results showed that autophagy inhibition elicits the switching of DNA repair from HR to the counter-productive NHEJ process, leading to sensitisation of BRCA-WT breast cancers to PARPi treatment.

The combination of talazoparib and chloroquine was also tested for its efficacy in HR-deficient BRCA1-KO and ATG5-KD MCF-7 cells. Our result showed that BRCA1-KO and ATG5-KD MCF-7 cells *vis-à-vis* control cells were more sensitive to talazoparib alone, which is correlated with known HR-deficiency in these cells (clonogenic and sub-G1 assay; Supplementary Fig. S13A–C). Further, cotreatment of talazoparib and CQ caused higher sensitisation in control cells, while the effect of sensitisation is marginal in BRCA1-KO and ATG5-KD MCF-7 cells (Supplementary Fig. S13A–C). Besides, 53BP1 foci formation corroborated with cell death in the respective cell lines in response to combination treatment (Supplementary Figs. S11A–D and S13A–C). Together, these results suggested that autophagy plays predominant role in control MCF-7 cells while the autophagy effect is marginal in HR-deficient BRCA1-KO and ATG5-KD MCF-7 cells in response to combination treatment.

In addition, we performed experiments on four other breast cancer cell lines: MDA-MB-453, T47-D, SKBR-3 and MDA-MB-468, showing varied sensitivities to talazoparib as indicated in our experiments as well as by the dataset obtained from ‘Genomics of drug sensitivities in cancer’ (https://www.cancerrxgene.org/; Supplementary Fig. S14A). T47-D and MDA-MB-453 cells were reported here as relatively resistant to talazoparib. Our result also showed that T47-D, MDA-MB-453 and SKBR-3, amongst the four breast cancer cell lines tested, are highly resistant to talazoparib treatment in cell viability and clonogenic assay (Supplementary Fig. 14B–G). Interestingly, the combination treatment of talazoparib and CQ further sensitised these cells (Supplementary Fig. 14B–G). Hence, talazoparib and CQ treatment are effective against multiple breast cancer cells.

### Efficacy and toxicity of talazoparib and CQ treatment in vivo xenograft tumour model

**Fig. 6:**
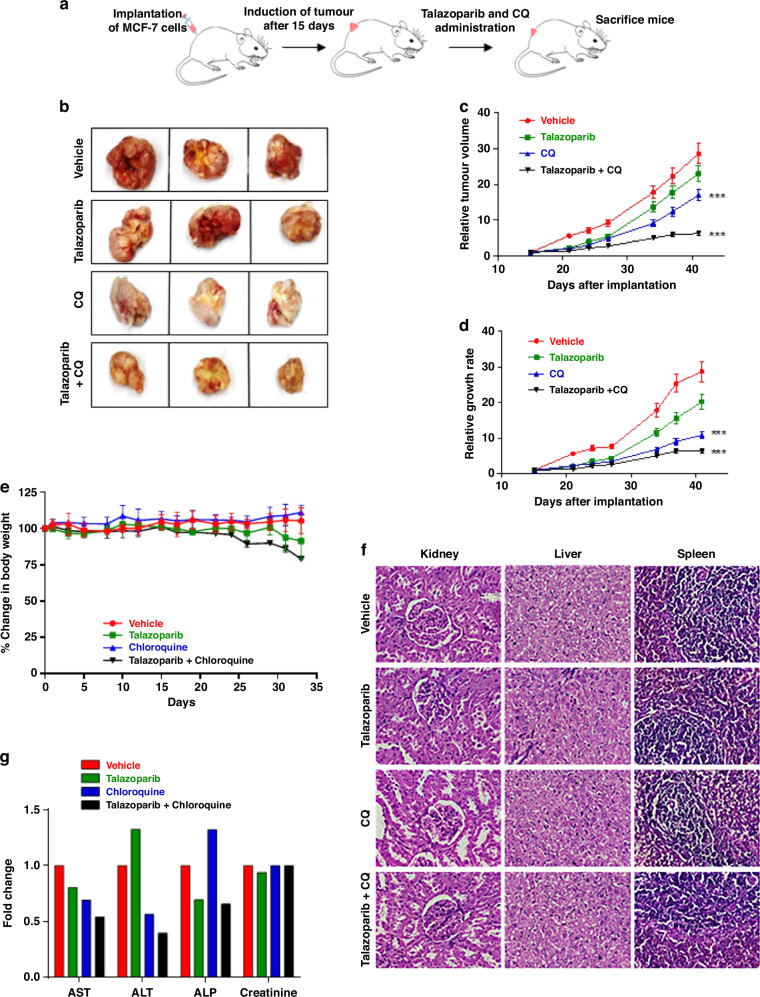
Pharmacological inhibition of autophagy sensitises BRCA1-WT MCF-7 tumour xenograft to PARPi treatment. **a** Schema of implantation of tumour xenograft, treatment regimen and determination of tumour burden in SCID mice. **b** SCID mice-bearing MCF-7 xenografts were treated with talazoparib alone, CQ alone or combination *p.o.* on alternate days (*n* = 6 for each group). Tumour volume was measured on the indicated days, and tumour volume and growth rate were assessed. **c**, **d** After 15 times of dosing, the mice were sacrificed, and tumours were dissected out and their weight and volume was assessed. **e** Normal non-tumour-bearing SCID mice were administered the same dosing indicated for the tumour-bearing xenograft mouse models, and the percentage change in the body weight was recorded on alternated days (*n* = 3 per group). Percent change in the body weight of the animal from day 1 of treatment was plotted against days of treatment. **f**, **g** After 15 dosings of the indicated treatments, mice were sacrificed following euthanasia, blood was drawn, clotted and serum was obtained for assessing the biochemical parameters. Liver, kidney and spleen were harvested upon sacrifice and were analysed by haematoxylin and eosin staining for histopathological changes under the microscope. Representative histology images are shown. Average readings of the biochemical parameter analysed were compared with the vehicle-treated group, and fold change obtained was plotted (*n* = 3 per group). For (**c**, **d**) values indicated are mean ± S.E.M. ***P < 0.001 compared to the vehicle-treated group.

We further evaluated the effect of the combination of talazoparib and CQ against xenograft breast tumour (BRCA-WT) in SCID mice. Combination treatment of talazoparib and CQ (15 doses, 30 days), was effective in reducing the tumour volume and growth rate in comparison to PARPi or CQ alone treated groups (Fig. 6a–d). Furthermore, our result also showed that the drug combination was well tolerated, and no deaths were observed during the experimental period (45 days), leading to survival being 100 % in all the groups.

Besides, we performed cell viability and clonogenic assays to determine toxicity to the non-malignant MCF-10A cells in response to talazoparib and CQ treatment. The results of cell viability assay at 72 h, as well as the long-term clonogenic assay, indicated significantly lower toxicity to non-malignant MCF-10A cells than MCF-7 cells (Supplementary Fig. S15A–D). We also analysed serum concentration of talazoparib at 2, 6 and 24 h after oral gavage of this drug (one dose of 2 mg/kg body weight). The serum level of talazoparib was assessed by the reported protocol.^26^ In this regard, we found that serum talazoparib concentration was ~25 nM at 2 h, which progressively reduced further with time (Supplementary Fig. S16).

Tolerability and toxicity associated with talazoparib and CQ treatment was also investigated by measuring (a) body weight on alternate days, (b) serum biochemical parameters for liver and kidney functions and (c) histopathological changes in the liver, kidney and spleen (Fig. 6e–g). Our analyses indicated no significant toxicity is associated with any of the tested treatments (Fig. 6e–g). Besides, we found that CQ treatment is well tolerated while talazoparib alone and combination treatment led to a drop in body weight (Fig. 6e), which was later recovered in another two weeks of non-treatment period. It is reported that ~20% loss in body weight is acceptable.^48^ Together, our results showed that although 25 nM serum concentration of talazoparib alone was less effective, a combination treatment of talazoparib and CQ was significantly effective in reducing BRCA1-WT breast xenograft tumour (Fig. 6a–d) with no or minimum toxicity. In this regard, a better formulation of talazoparib is warranted for further enhancing the therapeutic efficacy of the talazoparib.

## Discussion

PARPi monotherapy is not very effective in patients with HR-proficient breast cancers. A therapeutic strategy, therefore, is yet to be identified that is broadly applicable to all patients, irrespective of BRCA status, and for the tumours resistant to PARP inhibitors. In the current study, we demonstrated that the inhibition of autophagy greatly sensitises BRCA-WT breast cancers to talazoparib treatment. Autophagy has been the source of chemoresistance to several drugs. Some reports have tried to link the PARP activity or signalling with autophagy. It was earlier reported that the hyperactivation of PARP during DNA damage leads to a starvation-like situation in cells leading to autophagy.^49^ Intriguingly, we found that PARP inhibition with talazoparib led to upregulation of autophagy initiation factors and autophagic flux in the breast cancer cell lines tested. Certain drugs can only initiate autophagosome formation in the cells but their removal by lysosomes is abrogated. However, with our time-course experiments, we proved that autophagosome formation and its fusion with lysosomes was established upon talazoparib treatment.

To strengthen our findings to establish that autophagy was indeed playing a role in the de novo resistance to PARPi, we systematically depleted ATG5, p62/SQSTM1 and LAMP1 gene products essential at different stages of the autophagic pathway. We found significant sensitization of PARPi affected cell death in these cells, highlighting the pivotal role of autophagy in de novo resistance in BRCA-WT breast cancer cells to PARPi treatment. In response to IR and etoposide treatment, defective autophagy is linked to CHK1 degradation and abrogation of RAD51-loading.^22^ In ATG7^−/−^ and ATG5^−/−^ cells, HR is significantly suppressed and this switches DNA repair to NHEJ process, leading to genomic instability and cell death.^22,50^ In another independent study, it was reported that NHEJ plays a critical role in inducing cell death in HR-deficient ovarian cancer cells in response to PARPi treatment.^21^ Further, a recent study demonstrated that suppression of HRR by depletion of RAD51C and XRCC2 led to an increase in the NHEJ activity, genomic instability and cell death in response to PARPi.^51^ However, it is not yet known whether autophagy is linked to activation of HR and suppression of NHEJ, contributing to de novo resistance to PARPi in BRCA-WT breast cancers. In this study, we showed that autophagy inhibition, either by genetic targeting or pharmacological inhibition, indeed, led to the switching of the repair process to deleterious NHEJ process, as evident from the enhanced 53BP1 foci in talazoparib-treated cells. Further, autophagy inhibition led to extensive mitotic catastrophe related cell death in talazoparib-treated breast cancer cells. Taken together, we established that autophagy plays a crucial role in favouring HR at the expense of NHEJ to promote de novo resistance in BRCA-WT breast cancer cells.

In conclusion, in this study, we demonstrated that autophagy activation leads to de novo resistance to PARP inhibitors in BRCA-WT HR-proficient breast cancers. Therefore, (a) pharmacological targeting of autophagy along with PARP inhibitor for the majority of HR-proficient breast cancer cases or (b) targeting cancers with a genetic deficiency in autophagy with talazoparib would both lead to better therapeutic outcomes in HR-proficient cancer cells. Our findings demonstrate the potential benefits of PARPi and autophagy inhibitor combinatorial therapy and also propose to broaden the scope of the therapy to a larger population of patients with HR-proficient breast cancers and for tumours resistant to PARPi.

## Supplementary information

Supplemental Material

## Data Availability

The authors agree to make the data in this paper publicly available on genuine request.
