# Supplementary material for: Targeting autophagy reverses de novo resistance in homologous recombination repair proficient breast cancers to PARP inhibition
Source: Br J Cancer. 2021 Jan 21;124(7):1260–74. doi: 10.1038/s41416-020-01238-0 (PMC8007595; doi:10.1038/s41416-020-01238-0)
Supplement: Supplementary file 1 — Supplemental Material [file 41416_2020_1238_MOESM1_ESM.docx]

**Supplementary Information**

**Targetting autophagy reverses *de novo* resistance in homologous recombination repair proficient breast cancers to PARP inhibition**

Ganesh Pai Bellare^1,2^, Bhaskar Saha^1^, Birija Sankar Patro^1,2 *^

^1^Bio-Organic Division, Bhabha Atomic Research Centre, Trombay, Mumbai-400085, India, ^2^Homi Bhabha National Institute, Anushaktinagar, Mumbai-400094, India.

***
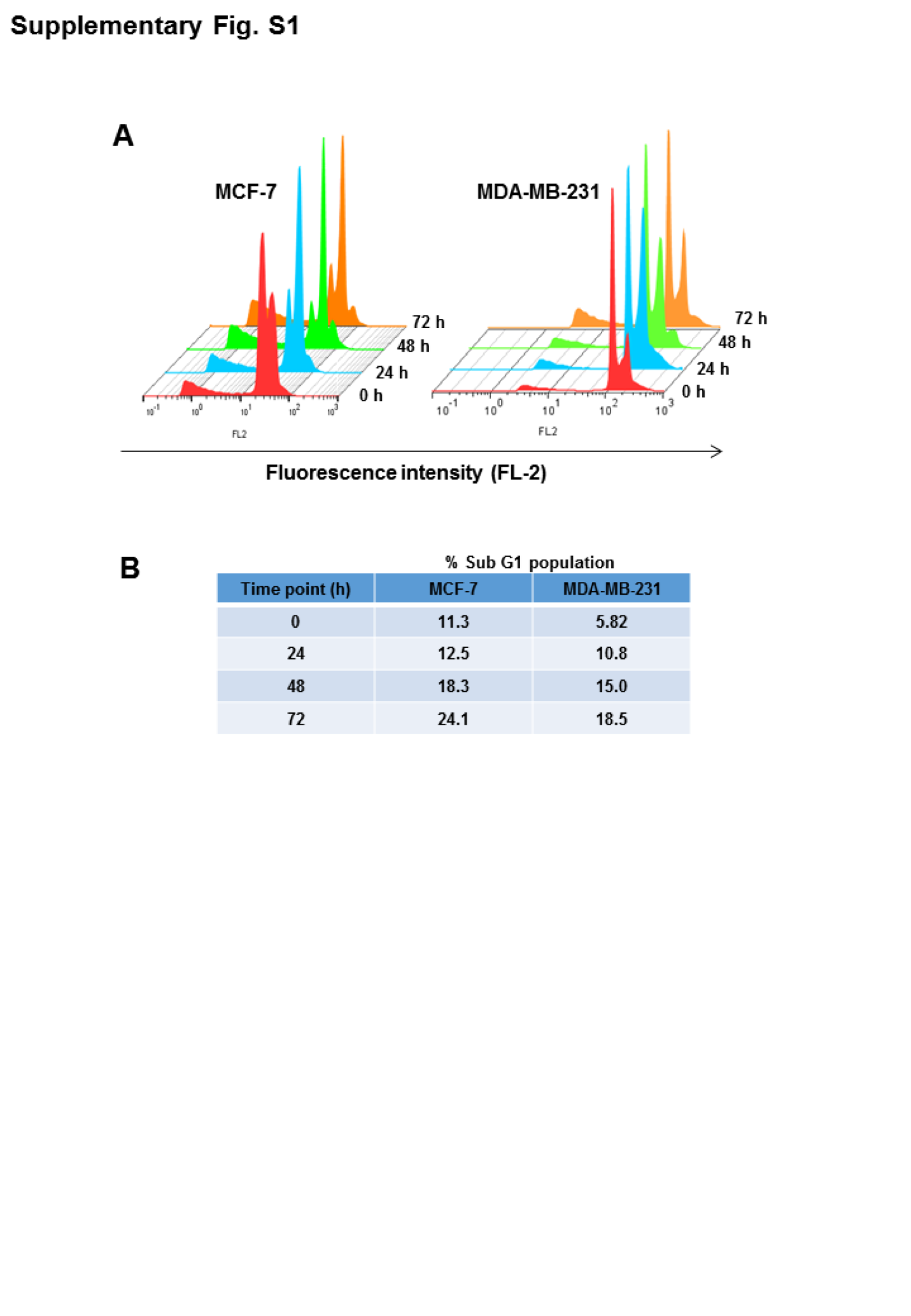
***

***Supplementary Figure S1: Talazoparib induces cell cycle arrest and cell death in MCF-7 and MDA-MB-231 breast cancer cell lines*** (A) MCF-7 and MDA-MB-231 cells were incubated with 200 nM talazoparib for 24 h, 48 h and 72 h. Cell cycle analysis and subG1 analysis were performed by flow cytometry acquiring the readout intensities of propidium iodide staining in FL2 and FL3 channels. FL2 channel readings in the logarithmic scale were used to assess sub-G1 population while FL3A channel on a linear scale was used for cell cycle analysis by Flow-Jo software (see Figure 1E). (B) Quantification of sub-G1 population at the indicated time points is provided.

***
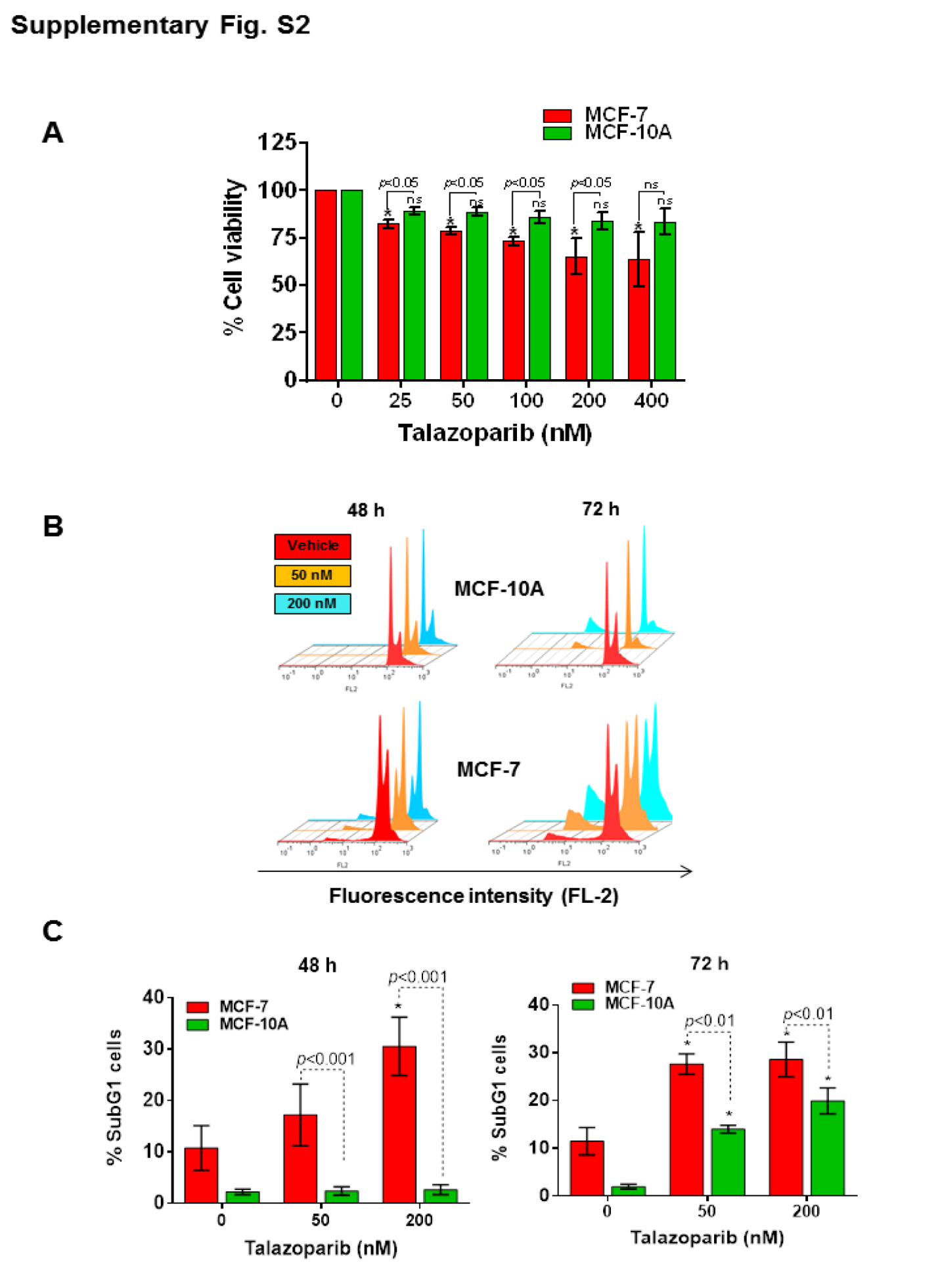
Supplementary Figure S2: Effect of talazoparib on MCF-7 and MCF-10A cell viability and apoptosis induction*** (A) MCF-7 and MCF-10A cells were treated with increasing concentrations of talazoparib for 72 h duration and the cell viability was assessed by MTT assay. (n=3) (B, C) MCF-7 and MCF-10A cells were treated with indicated concentrations of talazoparib for 72 h with the indicated concentrations and the apoptotic sub-G1 population was assessed by flow cytometry. Profiles and their respective quantification data are shown. (n=3). Values indicated are mean ± S.E.M. **p*<0.05 compared to untreated control in th respective cell line.

***
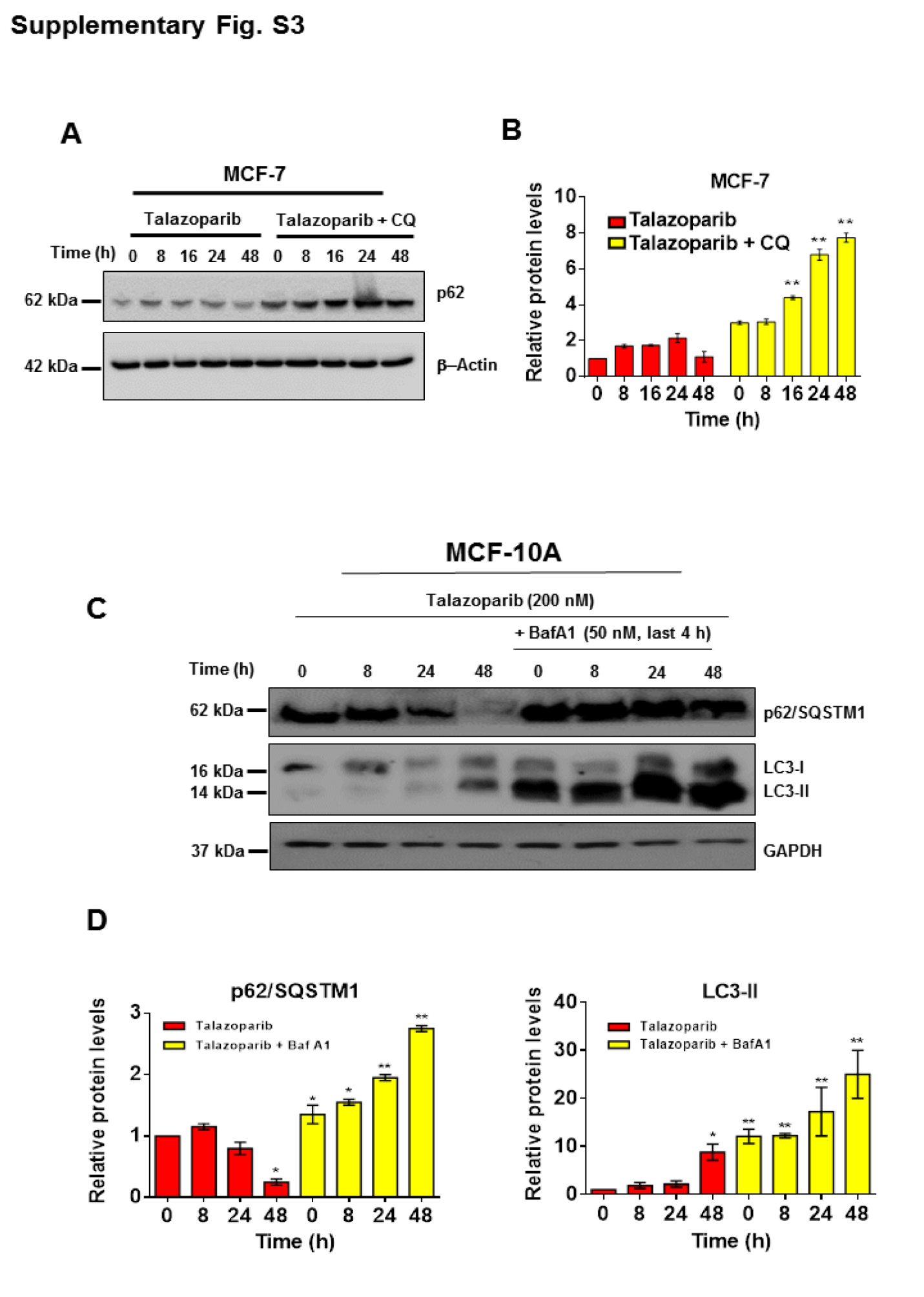
Supplementary figure S3: Talazoparib induces autophagic flux in both MCF-10A and MCF-7 cells.*** (A, B) MCF-7 cells were treated either with talazoparib (200 nM) or the combination of talazoparib (200 nM) and CQ (10 μM) for indicated time and immunoblotting assay was performed to check the level of p62/SQSTM1 protein as a surrogate to assess autophagic flux in MCF-7 cells. (C, D) MCF-10A cells were treated with talazoparib (200 nM) in the absence or presence of BafA1 (50 nM) for indicated time and immunoblot analysis was performed to assess autophagic flux. Bafilomycin A1 (50 nM) was added at the last 4 hours before the indicated time point. GAPDH was used as the loading control. In western blots, quantification of the band intensity was done by using ImageJ (v1.51j8). Relative protein levels were normalized with respect to the GAPDH levels and quantified with respect to the 0 hour treatment. Values indicated in the graph are mean ± S.E.M. **p*<0.05 and ***p*<0.01 compared to 0 h control.

***
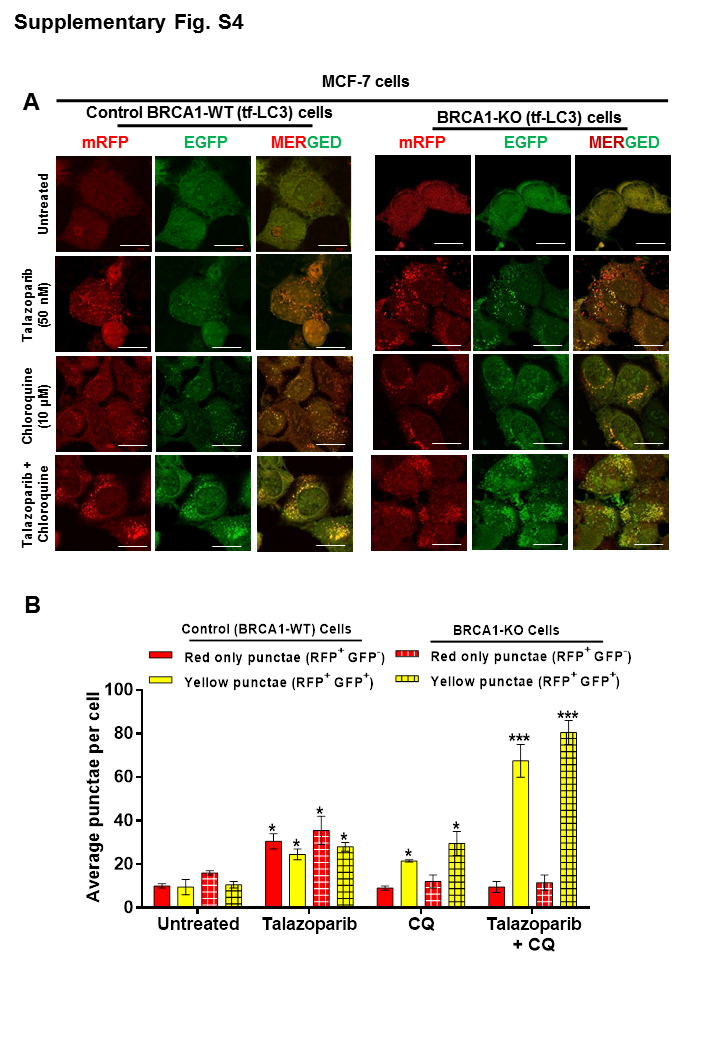
***

***Supplementary Figure S4.*** ***Comparison of autophagic flux in control and BRCA1-KO MCF-7 cell line.*** (A, B) Control and BRCA1-KO MCF-7 cells were treated with talazoparib (50 nM), chloroquine (10 µM) or their combination for 48 h and the autophagic flux was assessed by tf-LC3 assay by confocal microsocpy. Representative images are shown. Scale bar 20 μm. Red only (RFP^+^ GFP^­^) and yellow punctae (RFP^+^ GFP^+^) in cells were manually quantified from the merged image and the average number of red only punctae (RFP^+^ GFP^­^) and yellow punctae (RFP^+^ GFP^+^) per cell were plotted. Values indicated are mean ± S.E.M. **p*<0.05 and ****p*<0.001 compared to respective parameter in the untreated group of the respective cell type. Comparison of red only (RFP^+^ GFP^­^) and yellow punctae (RFP^+^ GFP^+^) formation was not signifcantly different in control (BRCA1-WT) and BRCA1-KO MCF-7 cells in different treatment conditions.

***
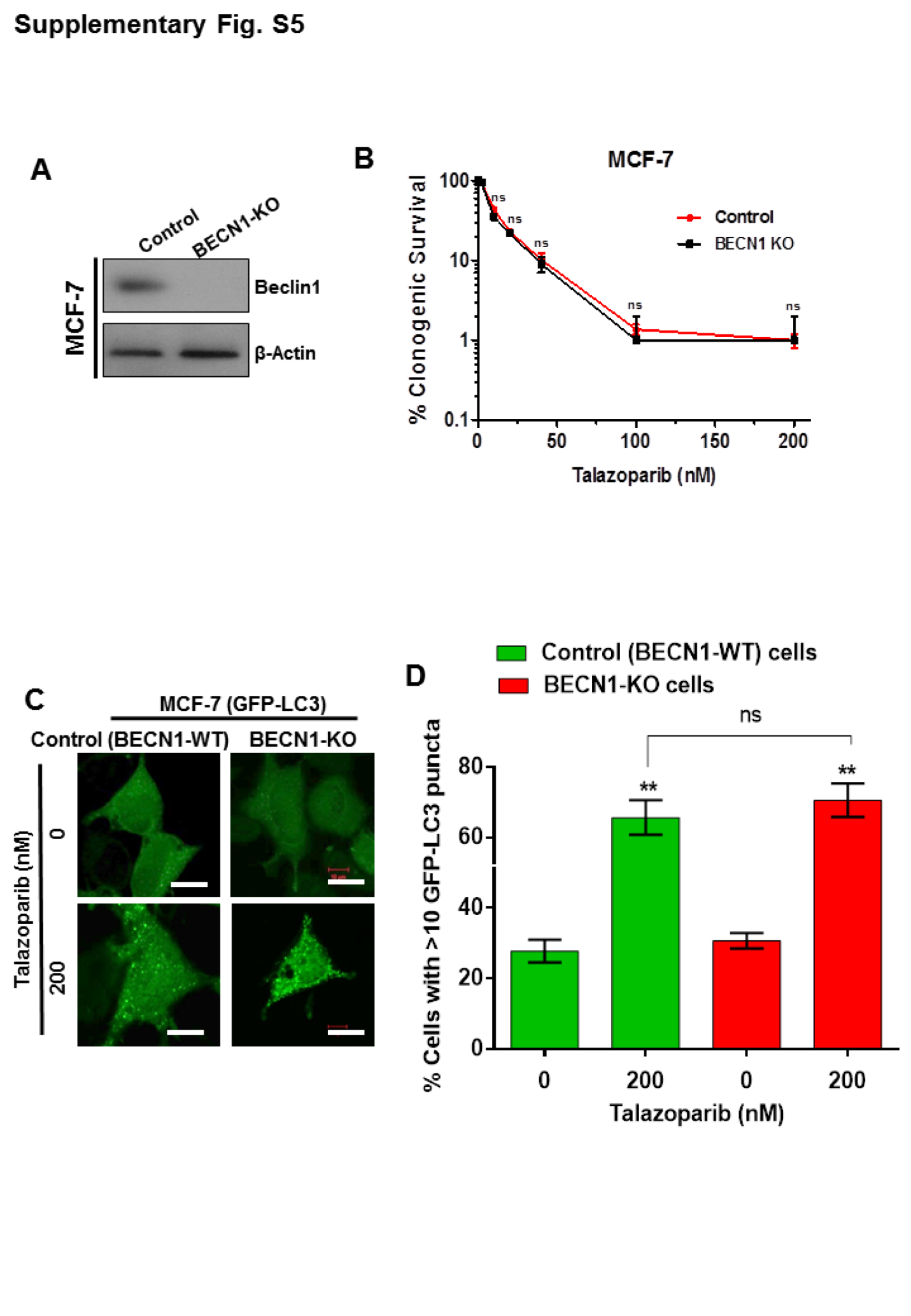
*** ***
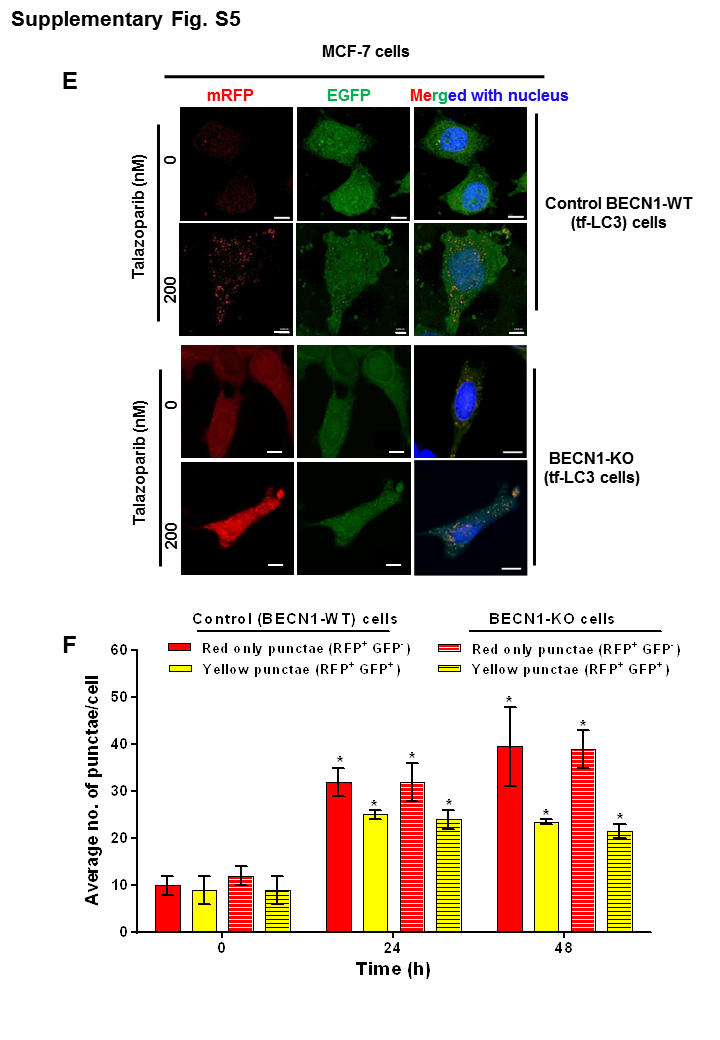
Supplementary figure S5: Talazoparib induces beclin1 independent autophagy in BRCA1-WT MCF-7 breast cancer cells.*** (A) Beclin1 (BECN1) knock-out cells were generated by using CRISPR-Cas9 double nickase system in BRCA1-WT MCF-7 cells. The levels of BECN1 protein in control and BECN1-KO cells was assessed by western blot analysis. (B) Control MCF-7 and BECN1-KO MCF-7 cells were treated with indicated concentrations of talazoparib for 6-8 days and their clonogenic potential was assessed. (C, D) Control and BECN1-KO MCF-7 cells stably expressing EGFP-LC3, were treated with talazoparib (200 nM) for 48 h and autophagosome formation was assessed by confocal microsocpy. Green GFP-LC3 punctae in cells were manually quantified and plotted. Scale bar 20 μm. (E) Control (BECN-WT) and BECN1-KO MCF-7 cells, stably expressing mRFP-EGFP-LC3, were treated with talazoparib (200 nM) for 48 h and autophagic flux was assessed by confocal microscopy. Representative images are shown. Red only (RFP^+^ GFP^­^) and yellow punctae (RFP^+^ GFP^+^) in cells were manually quantified from the merged image and the average number of red only punctae (RFP^+^ GFP^­^) and yellow punctae (RFP^+^ GFP^+^) per cell were plotted. Scale bar: 10 μm. Values indicated are mean ± S.E.M. (n=3). For D: ***p*<0.01 compared to vehicle control in the respective cell types. In B and D: ns is not significantly different compared to respective treatment in control MCF-7 cells. For F: **p*<0.05 compared to respective parameter in the untreated group of the respective cell type. Comparison of red only (RFP^+^ GFP^­^) and yellow punctae (RFP^+^ GFP^+^) formation was not signifcantly different in control (BECN1-WT) and BECN1-KO cells in different treatment conditions.

***
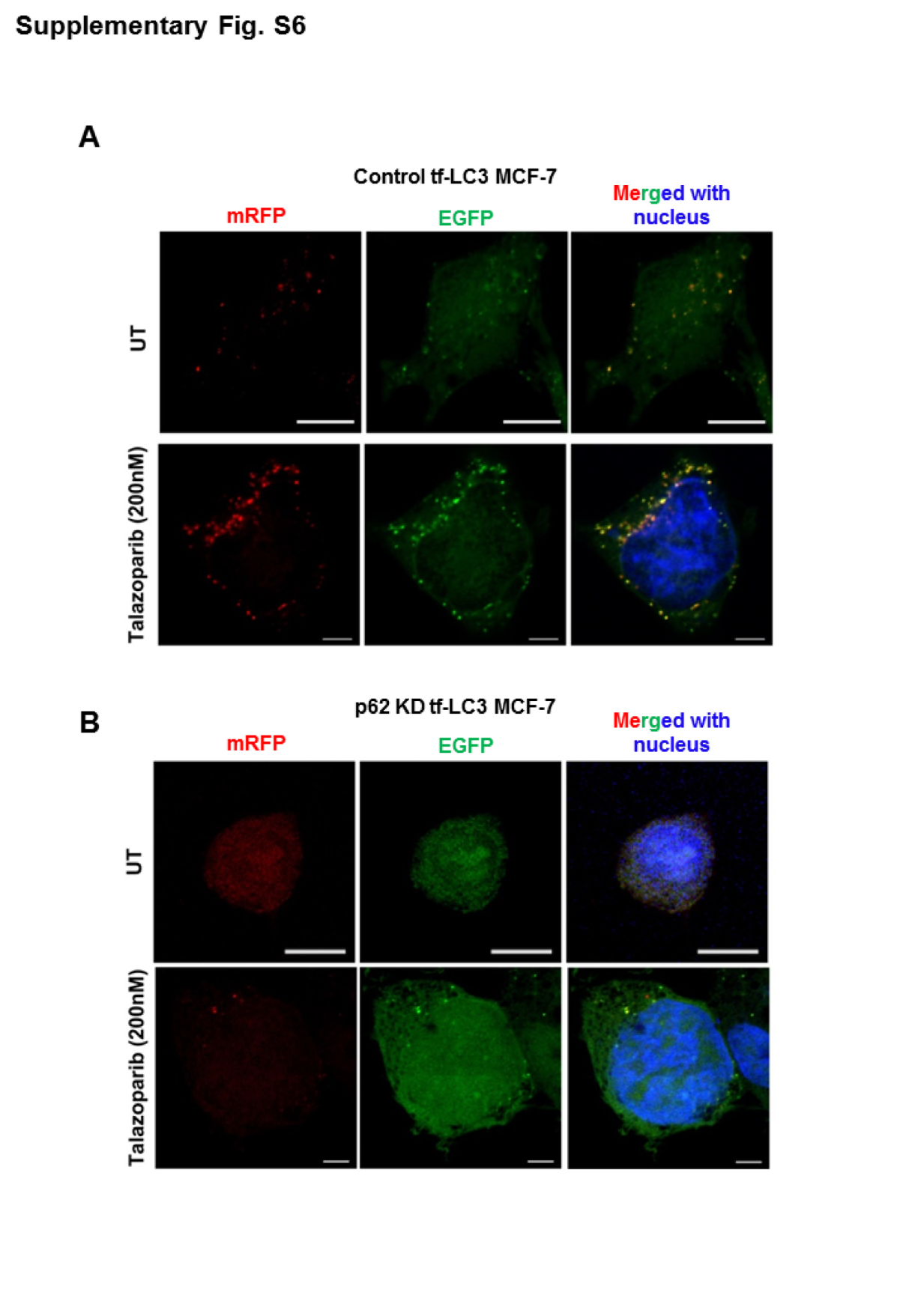
Supplementary figure S6: p62-KD MCF-7 cells are autophagy defective.*** (A, B). Control and p62-KD MCF-7 cells were treated with talazoparib (200 nM) for 48 h and autophagic flux was assessed visually by confocal microsocpy. Representative images are shown. Scale bar 20 μm.

***
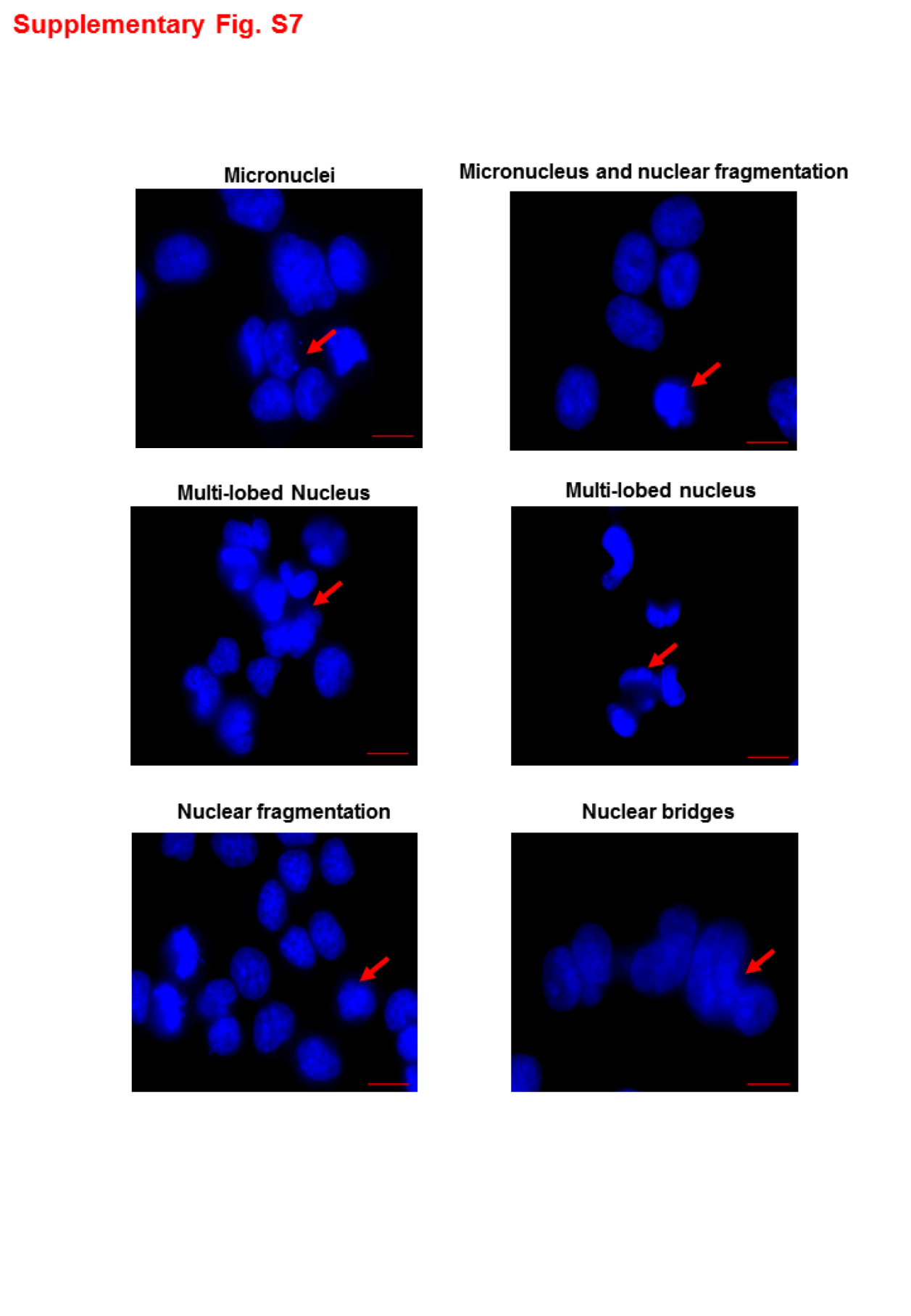
Supplementary Figure S7: Representative images of mitotic catastrophe events.*** Cells were treated with talazoparib for 48 h and events leading to mitotic catastrophe were assessed. Nuclei with micronuclei and abnormal features (multi-lobular, nuclear bridges, nuclear fragmentation) were considered as mitotic catastrophe positive. Scale bar 20 μm

***
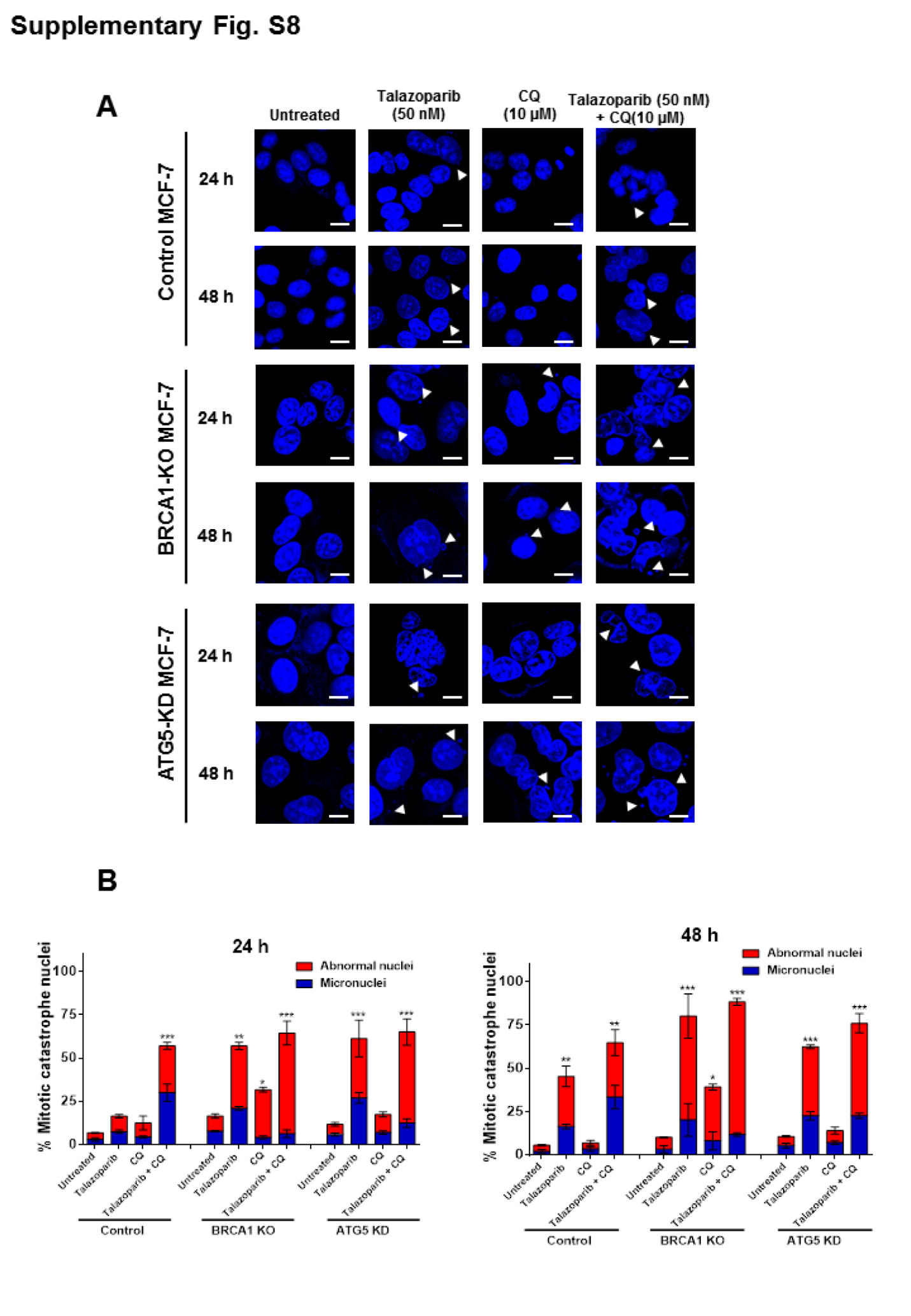
Supplementary figure S8: Pharmacological inhibition of autophagy induces mitotic catastrophe.***  (A-D) Control, BRCA1-KO and ATG5-KD MCF-7 cells were treated with talazoparib (50 nM), CQ (10 μM) or combination of talazoparib and CQ for 24 and 48 h and mitotic catastrophe events were assessed by confocal microscopy by staining the nucleus. Quantification was done by manual counting of mitotic catastrophe nuclei. Scale bar 20 μm. n=2 and the values indicated are mean ± S.E.M. **p*<0.05 and ****p*<0.001 compared to respective vehicle control of respective cell types.

***
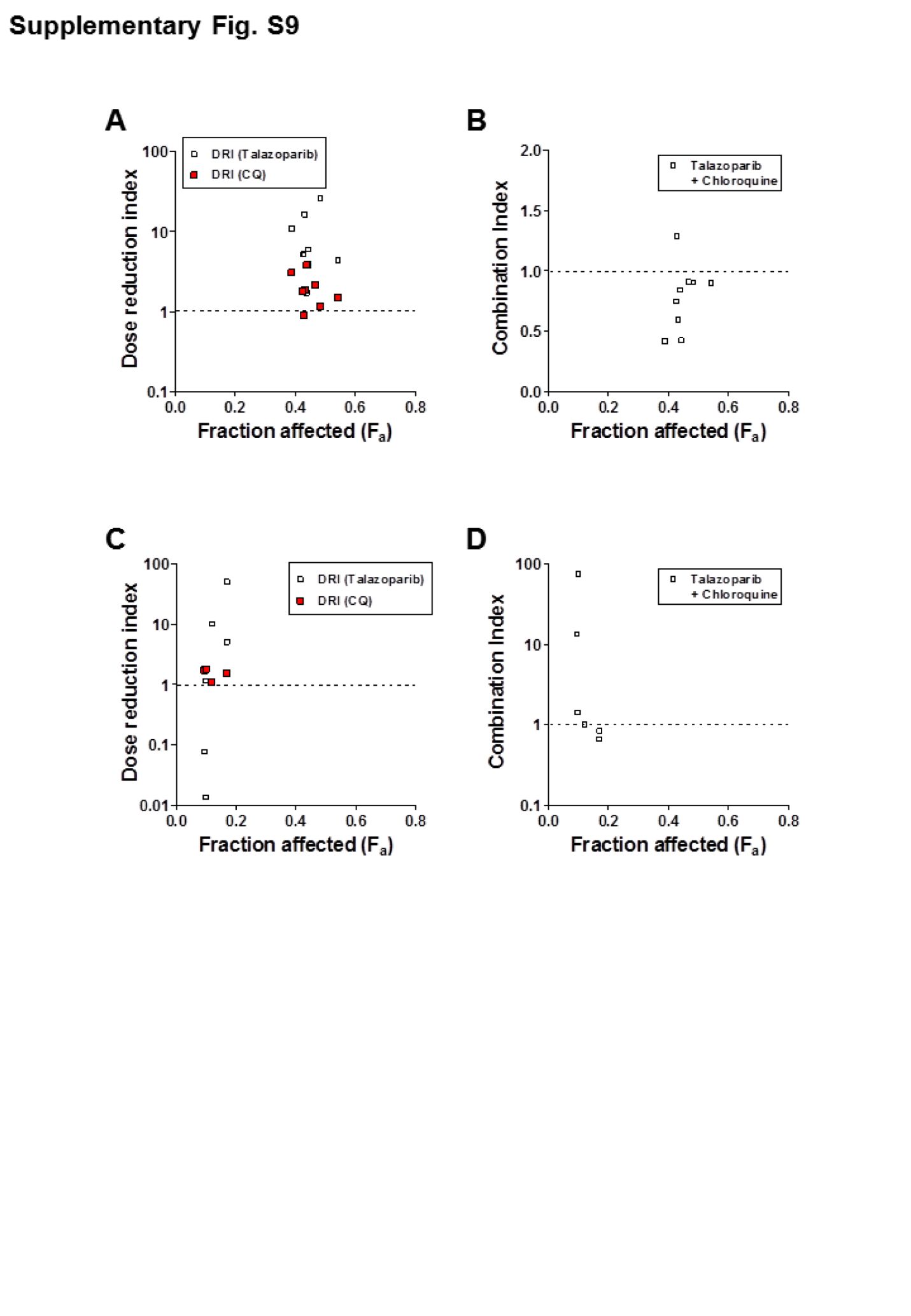
Supplementary figure S9: Combination index calculation by Compusyn software.*** The data for combination index analysis was obtained from sub-G1 analysis by flow cytometry. (A) Dose reduction index (DRI) was plotted against fraction affected (Fa, cell death induced) for MCF-7 cells treated with talazoparib, chloroquine and the combination. (B) Combination Index (CI) vs. fraction affected (Fa) graph of the Compusyn based analysis for the same dataset as (A). (C) Dose reduction index (DRI) was plotted against fraction affected (Fa) for MDA-MB-231 cells treated with talazoparib, chloroquine and the combination. (D) Combination Index (CI) vs. fraction affected (Fa) graph of the Compusyn based analysis for the same dataset as (C). A combination index of CI < 1 demonstrates pronounced synergism. Higher the DRI, better is the acitivity of the drug in combination compared to alone treatment.

***
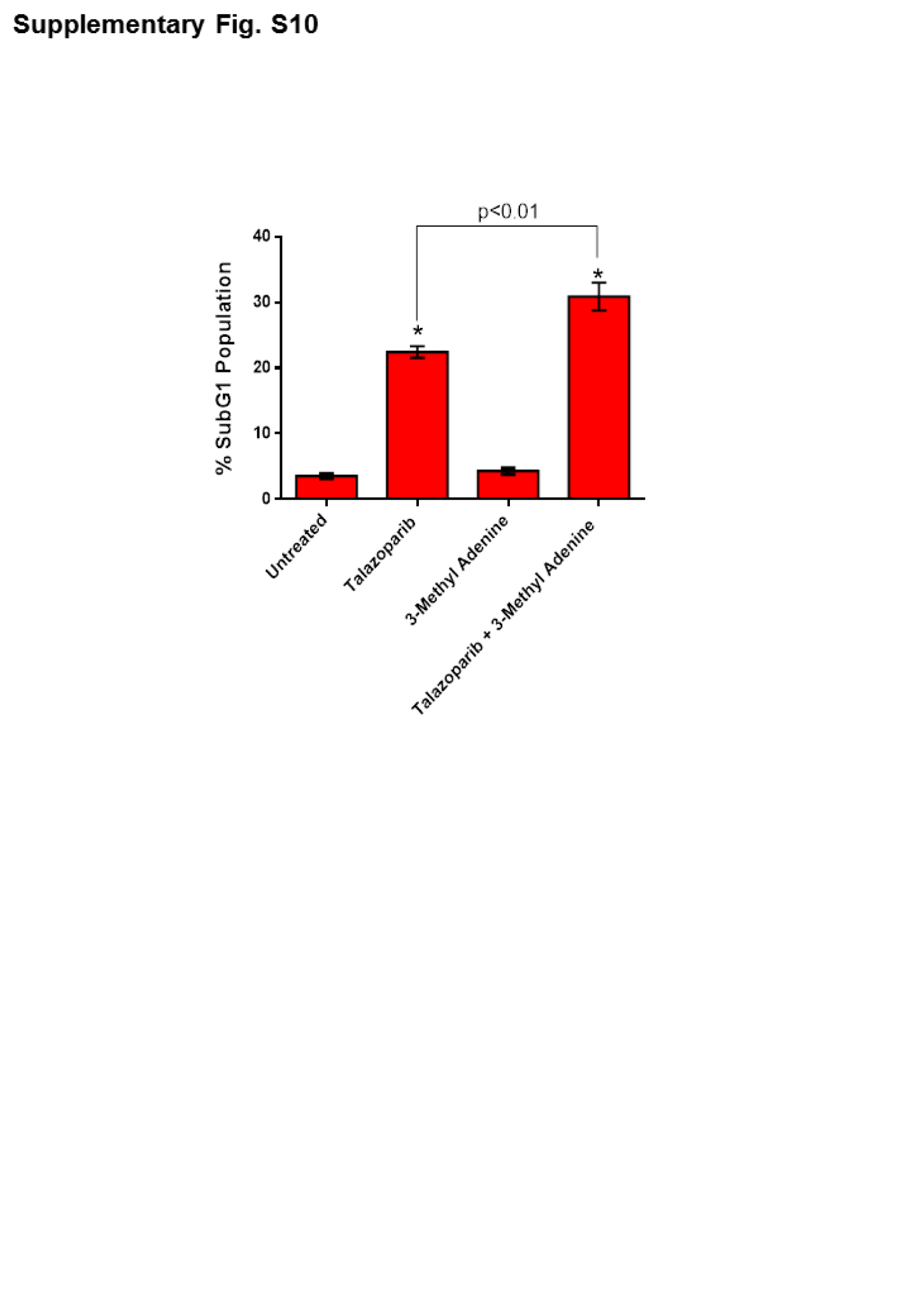
Supplementary figure S10: Pharmacological inhibition of autophagy sensitizes MCF-7 breast cancer to PARPi.*** MCF-7 cells were treated with talazoparib (200 nM), 3-methyl adenine (3-MA, 2.5 mM) or combination of talazoparib and 3-methyl adenine for 48 h and cell death was assessed by sub-G1 analysis using flow cytometry. Quantification was done using FlowJo software. All the determinations were made from 3 experiments, and the values indicated are mean ± S.E.M. **p*<0.05 compared to untreated control.

***
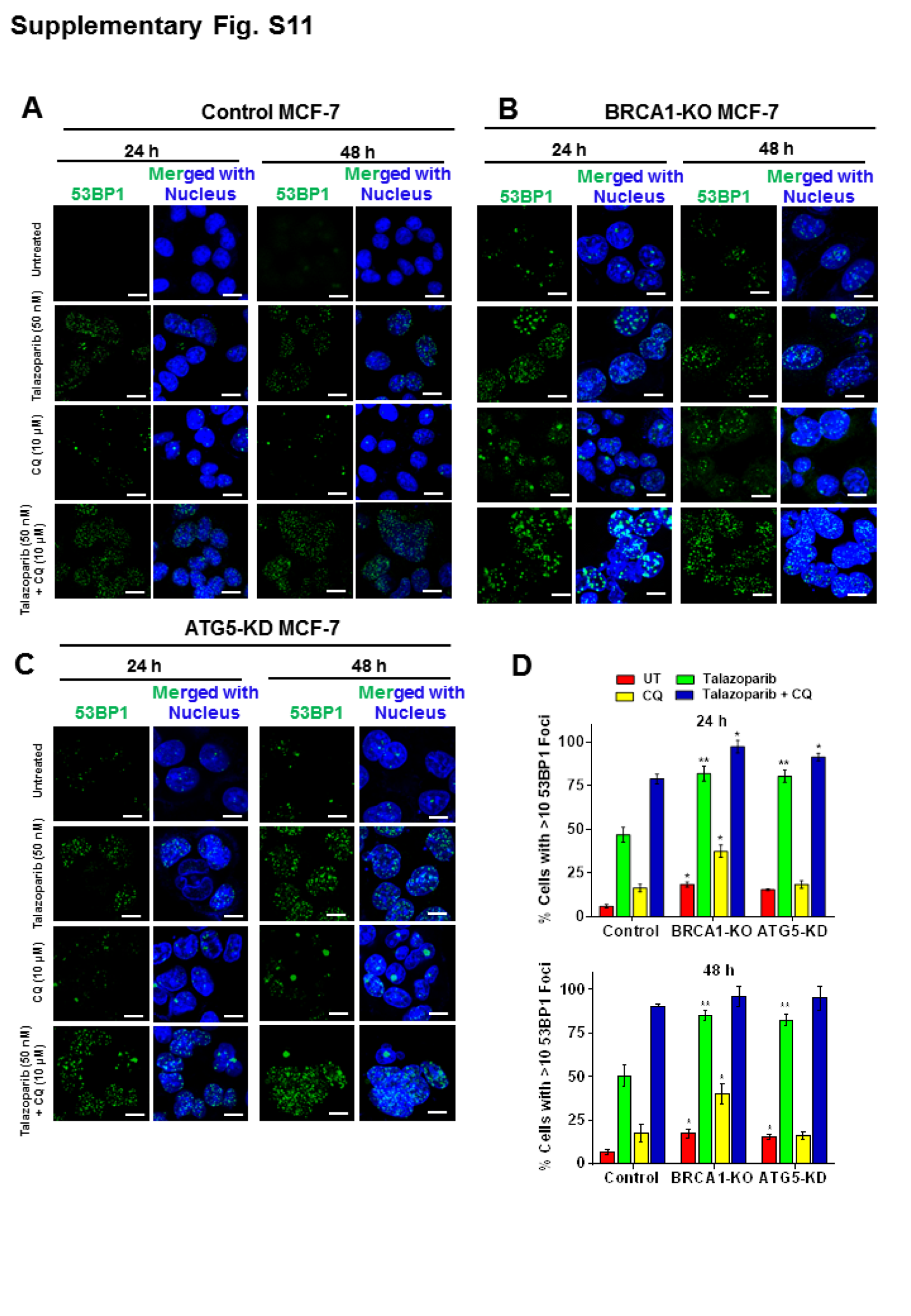
***

***Supplementary figure S11: 53BP1 foci formation under different treatment conditions in control, BRCA1 KO and ATG KD MCF-7 cells.*** (A, B, C) Control, BRCA1-KO, ATG-KD MCF-7 cells were treated with talazoparib (50 nM), CQ (10 µM) or the combination of talazoparib and CQ for 24 h and 48 h and the 53BP1 foci formation was assessed by immunofluorescence assay. Scale bar 20 μm. The samples were analysed by confocal microscopy and quantification was done by manually counting cells with >10 foci. (D) Quantification of cells having >10 53BP1 foci. n=2 for each experiment, values indicated are mean ± S.E.M. **p*<0.05 and ***p*<0.01 compared to untreated sample in control MCF-7 cells.


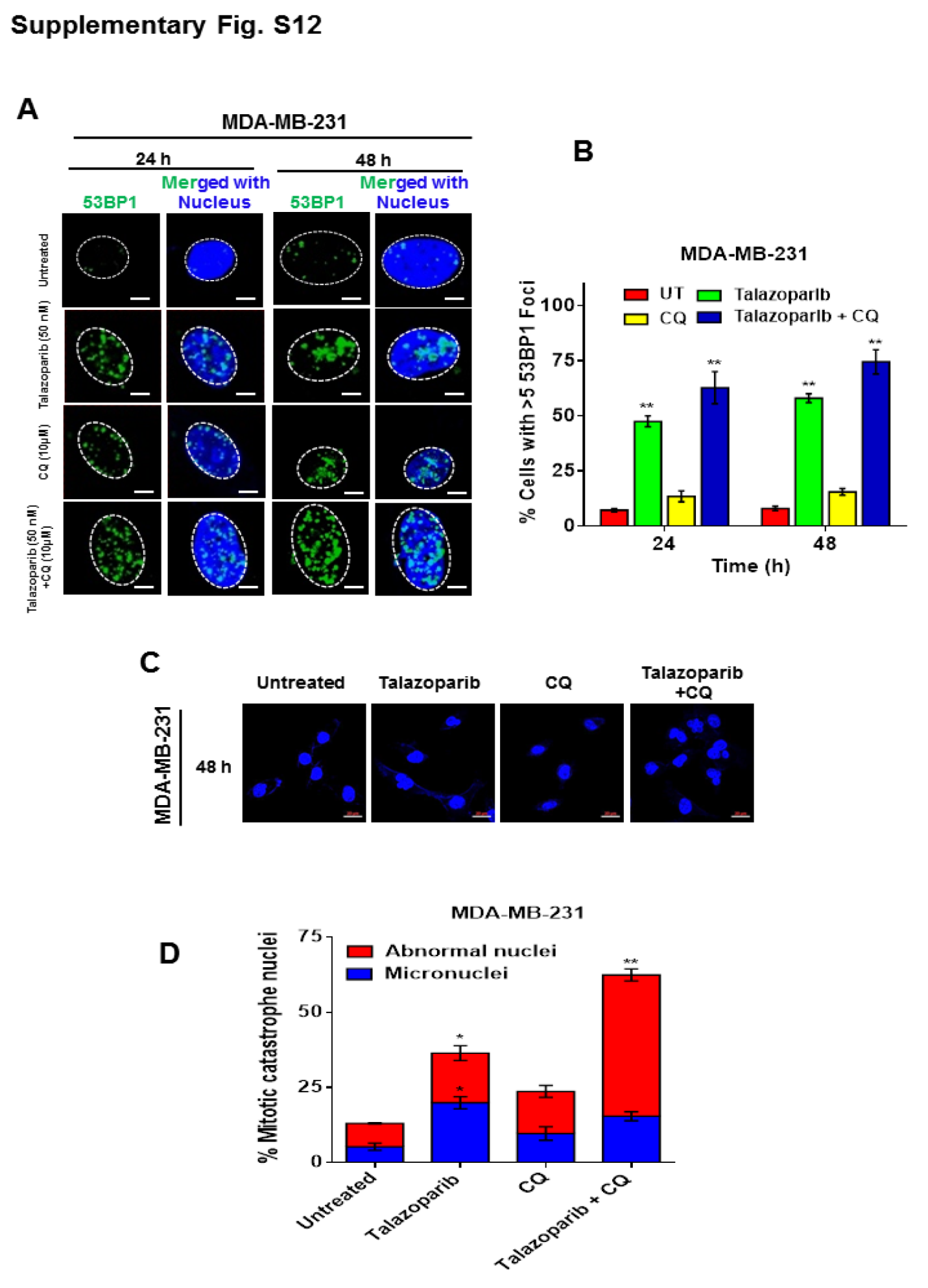


***Supplementary figure S12: 53BP1 foci formation and mitotic catastrophe in MDA-MB-231 cells in response to talazoparib and combination treatment.*** (A, B) MDA-MB-231 cells were treated with talazoparib (50 nM), CQ (10 µM) or the combination of talazoparib and CQ for 24 h and 48 h and the 53BP1 foci formation was assessed by immunofluorescence assay. (C, D) MCF-7 and MDA-MB-231 cells were treated with talazoparib (50 nM), CQ (10 μM) or combination of talazoparib and CQ for 24 and 48 h and mitotic catastrophe events were assessed by confocal microscopy by staining the nucleus. Quantification was done by manual counting of mitotic catastrophe nuclei. Scale bar 20 μm. n=2 and the values indicated are mean ± S.E.M. For B: ***p*<0.01 compared to respective vehicle control at respective time points, For D: **p*<0.05 and ***p*<0.01 compared to untreated.

**Supplementary Fig. S13**


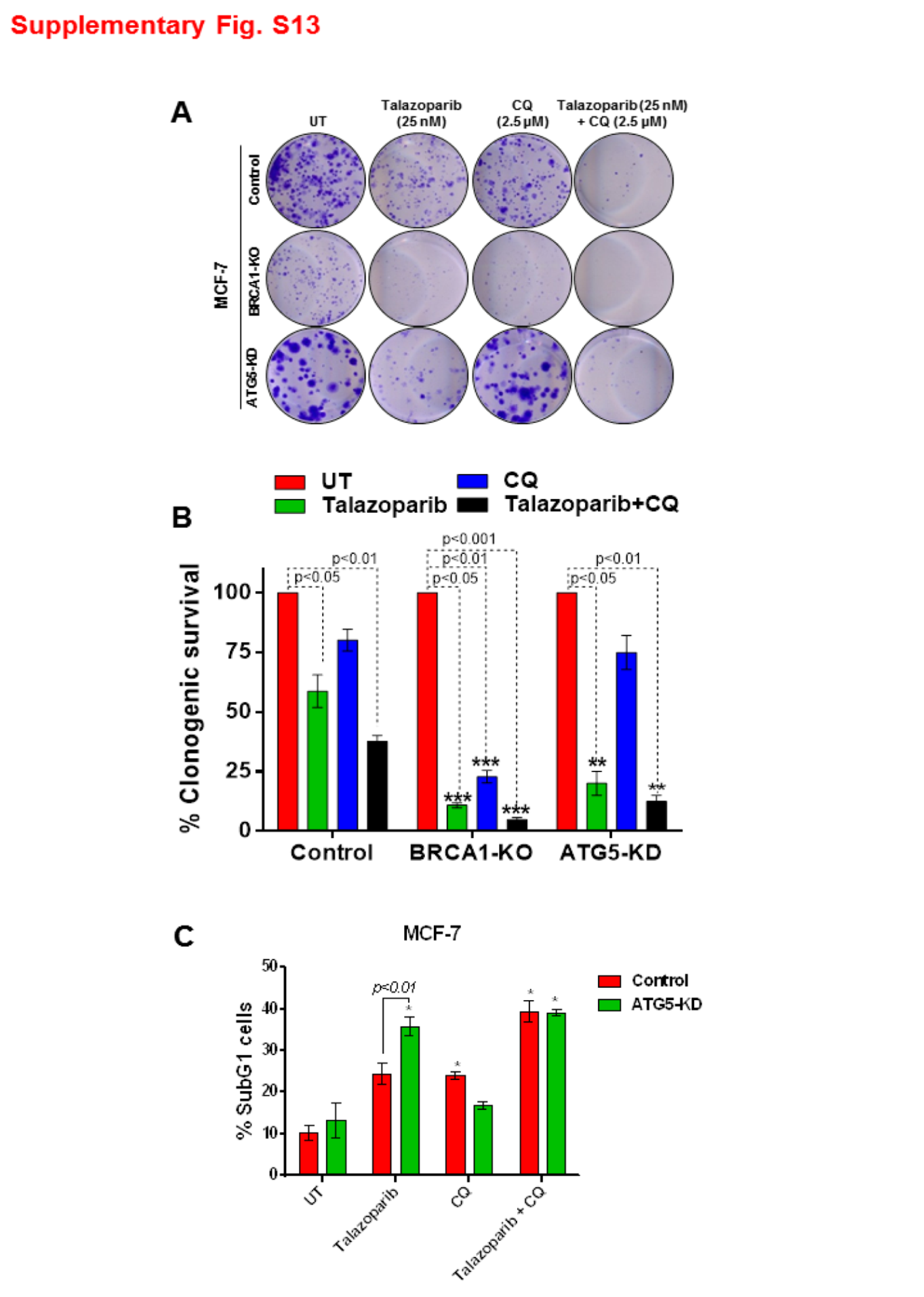


***Supplementary figure S13: Comparison of cell death induced by combnation of talazoparib and chloroquine in control, BRCA1-KO and ATG5-KD MCF-7 cells.*** (A,B) 300-500 cells of control, BRCA1-KO, ATG-KD MCF-7 cells were seeded in a 12 well plate and after overnight incubation, treated with talazoparib (25nM), chloroquine (2.5 µM) or the combination of these two drugs for 9 days and their clonogenic potential was assessed. The clonogenic survival was calculated with respect to the control and plotted. (n=3). (C) control and ATG-KD MCF-7 cells were seeded in a 6 well plate and after overnight incubation, treated with talazoparib (25nM), chloroquine (10 µM) or the combination and sub-G1 population was assessed at 72 h. Values indicated are mean ± S.E.M. For B: ****p*<0.01 compared to respective treatement in the control cells. For B: **p*<0.05 compared to respective untreated cells.

*
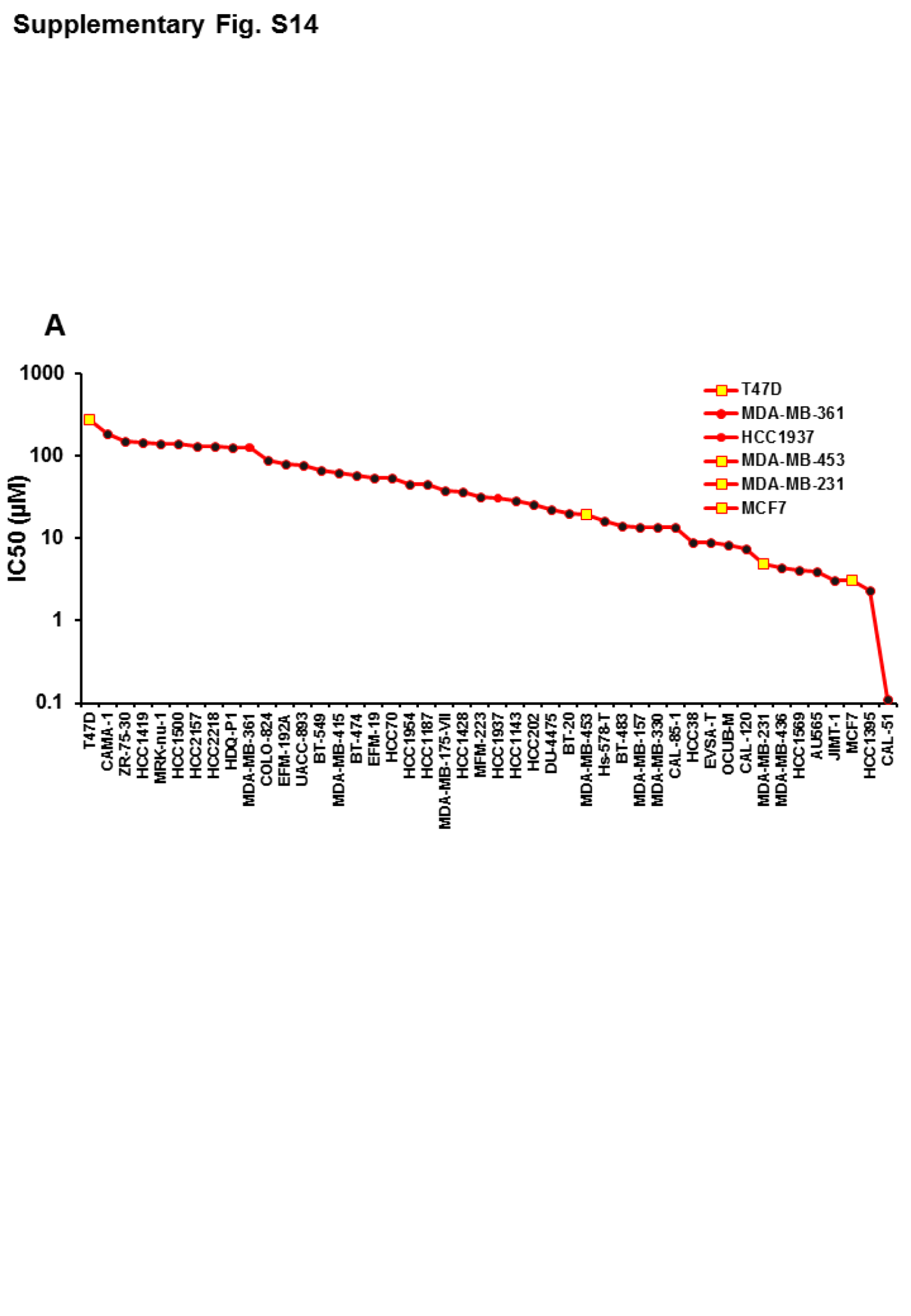
*


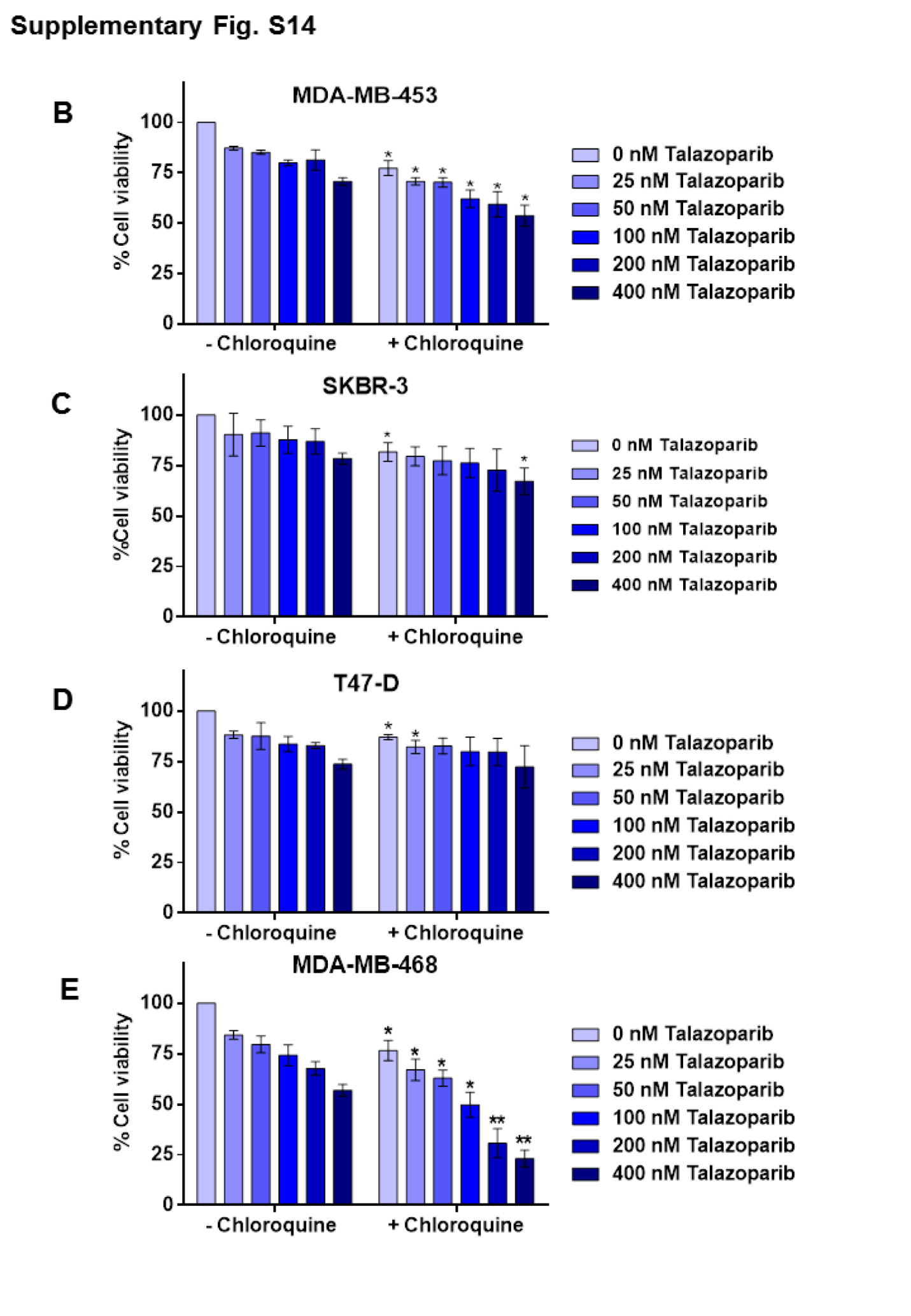


***
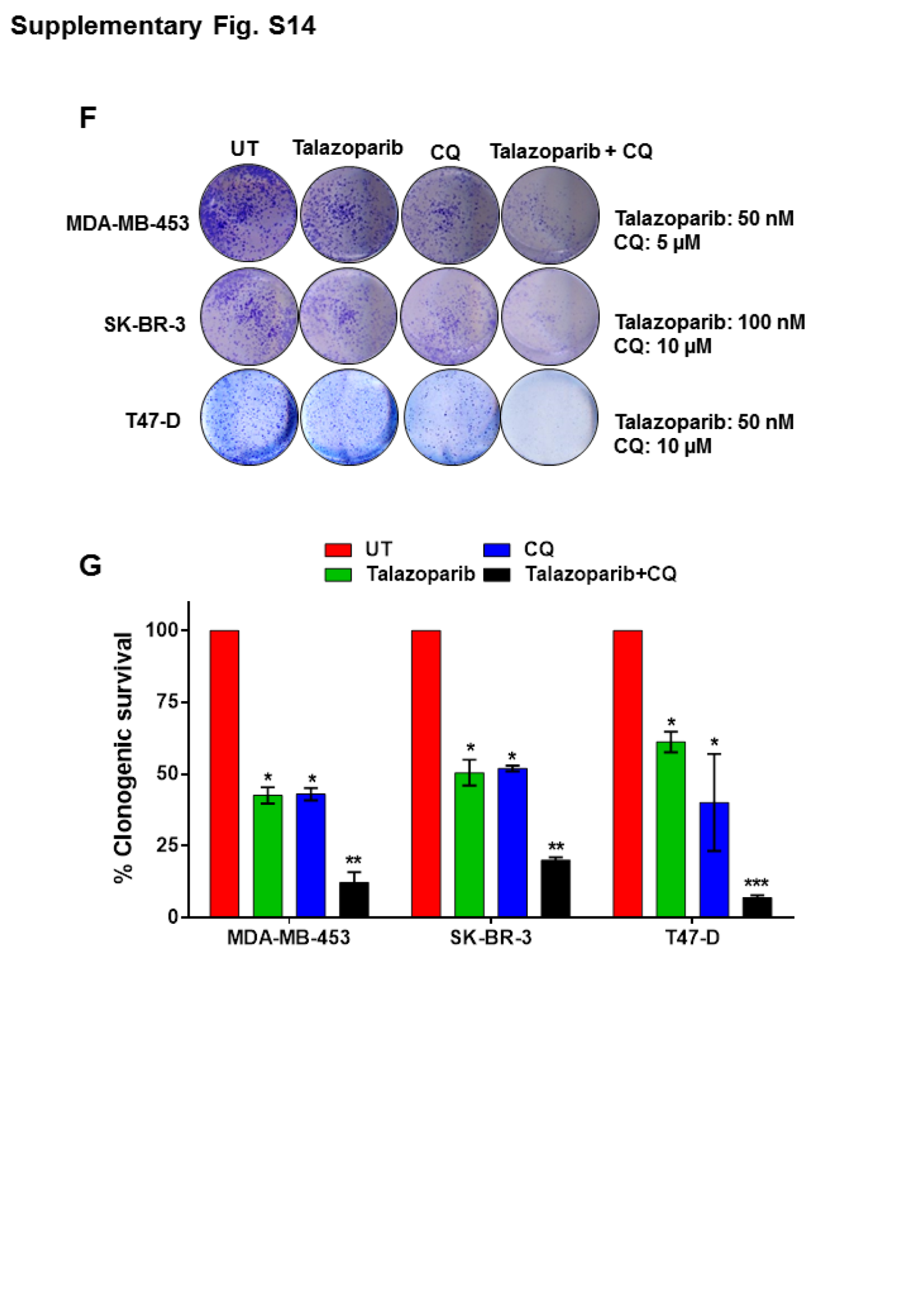
 Supplementary figure S14: Sensitivity of different breast cancer cell lines to the combination of talazoparib and chloroquine.*** (A) IC50 value data of talazoparib in different cell lines obtained from Genomics of drug sensitivities in cancer (GDSC) dataset 1. Cell lines used in this study are indicated in yellow boxes. (B-E) MDA-MB-453, SKBR-3, T47-D and MDA-MB-468 cells were treated with increasing concentrations of talazoparib in the absence or presence of chloroquine for 72 h duration to assess cell viability by MTT assay. (n=2) (F, G) Colony forming potential or clonogenic survival of MDA-MB-453, SKBR-3 and T47-D cells were assessed after treatment with indicated talazoparib and chloroquine concentrations. Colonies formed were fixed, stained and observed under microscope for counting. Percentage clonogenic survival with respect to the respective control group was plotted. (n=2). Values indicated are mean ± S.E.M. **p*<0.01, ***p*<0.01, ****p*<0.001 compared to the respective untreated group.

***
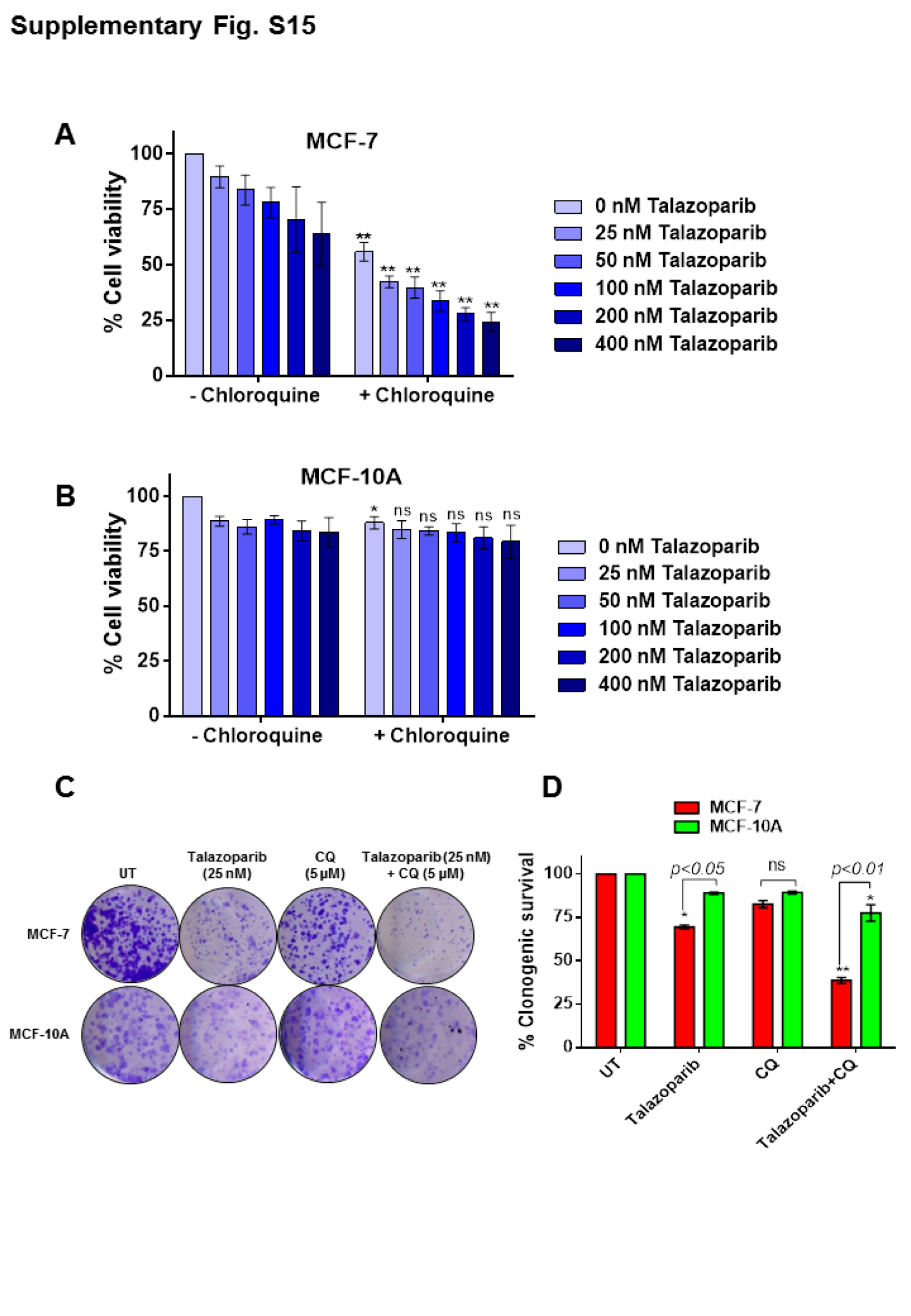
Supplementary figure S15: Toxicity effect of of the talazoparib and chloroquine combination on MCF-7 and MCF-10A cell lines.*** (A, B) MCF-7 and MCF-10A cells were treated with different concentrations of talazoparib in the presence or absence of chloroquine and their viability was assessed by MTT assay after 72 h. (C, D) MCF-7 and MCF-10A cells were incubated with talazoparib (25 nM), chloroquine (5 µM) or the combination of these two drugs for 9 days and their clonogenic potential was assessed. The clonogenic survival was calculated with respect to the control and plotted. (n=3 for each experiment). Values indicated are mean ± S.E.M. **p*<0.05, ns refers to no significance compared to vehicle control in (A) and (B). **p*<0.05, ***p*<0.01, compared to vehicle control in the respective cell lines.


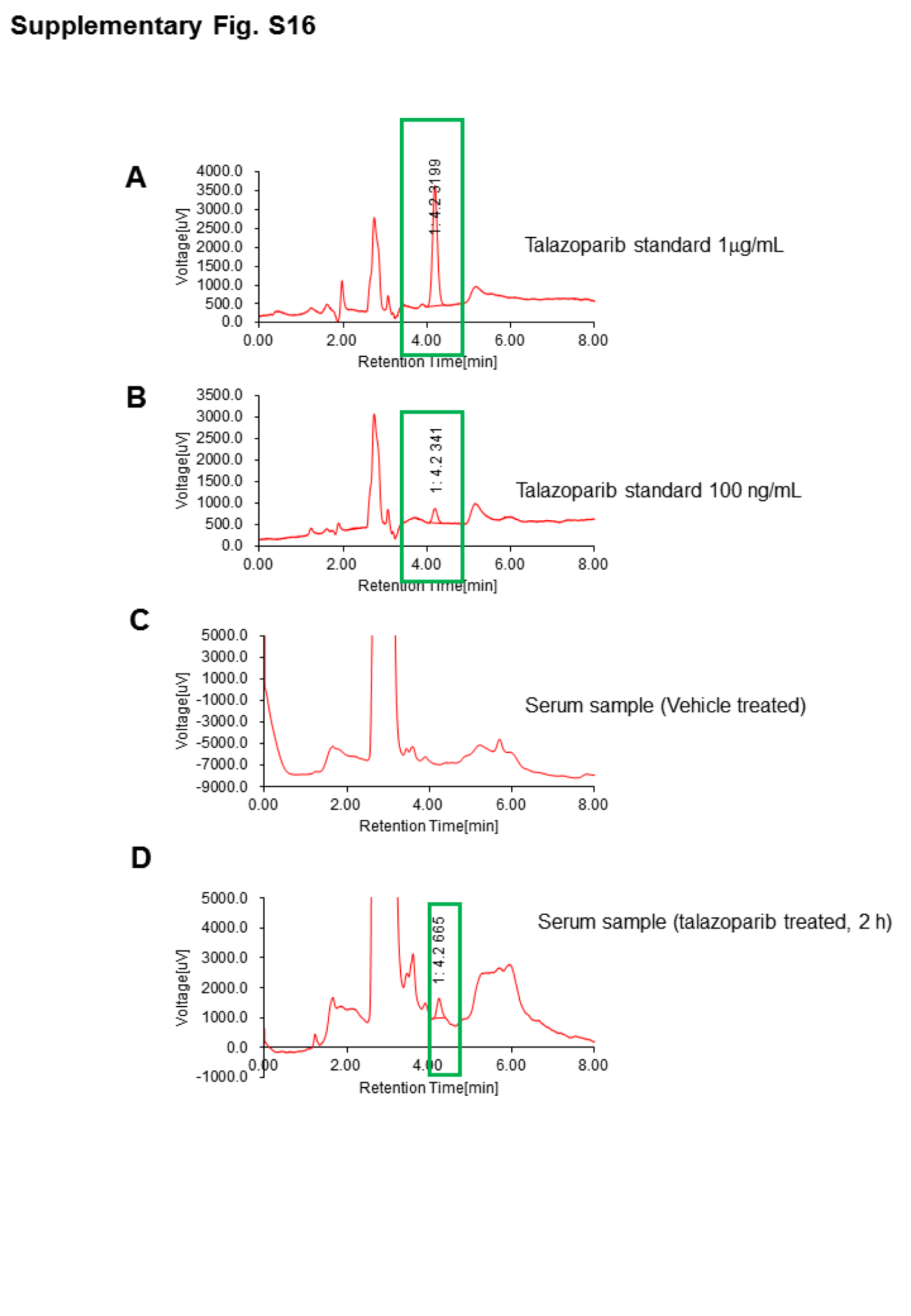


***Supplementary figure S16: HPLC profiles for detection of talazoparib in mice serum.***

SCID mice were given talazoparib (2 mg/kg body wt.) *per os*. After 2 h, mice were euthanised, sarcified and blood was drawn by cardiac puncture. The blood was clotted and and talazoparib in the serum was extracted as per protocol, mentioned in method section. Extracted samples were injected into HPLC column and profile obtained. Representative profiles are shown. (A) talazoparib standard (1µg/mL), (B) talazoparib standard (100 ng/mL), (C) vehicle treated samples and (D) 2 h talazoparib treated mice sample. Highlighted box represents talazoparib peak.
